# Finnish National Phenological Network 1997–2017: from observations to trend detection

**DOI:** 10.1007/s00484-020-01961-6

**Published:** 2020-07-06

**Authors:** Samuli Helama, Anne Tolvanen, Jouni Karhu, Jarmo Poikolainen, Eero Kubin

**Affiliations:** 1 Natural Resources Institute Finland, Ounasjoentie 6, 96200 Rovaniemi, Finland; 2 Natural Resources Institute Finland, University of Oulu, P.O. Box 413, 90014 Oulu, Finland

**Keywords:** Plant phenology, Advance of spring, Autumn phenology, Boreal ecosystems, Climate research

## Abstract

Plant phenological dataset collected at 42 sites across the mainland of Finland and covering the years 1997–2017 is presented and analysed for temporal trends. The dataset of *n* = 16,257 observations represents eleven plant species and fifteen phenological stages and results in forty different variables, i.e. phenophases. Trend analysis was carried out for *n* = 808 phenological time-series that contained at least 10 observations over the 21-year study period. A clear signal of advancing spring and early-summer phenology was detected, 3.4 days decade^−1^, demonstrated by a high proportion of negative trends for phenophases occurring in April through June. Latitudinal correlation indicated stronger signal of spring and early-summer phenology towards the northern part of the study region. The autumn signal was less consistent and showed larger within-site variations than those observed in other seasons. More than 60% of the dates based on single tree/monitoring square were exactly the same as the averages from multiple trees/monitoring squares within the site. In particular, the reliability of data on autumn phenology was increased by multiple observations per site. The network is no longer active.

**Electronic supplementary material:**

The online version of this article (10.1007/s00484-020-01961-6) contains supplementary material, which is available to authorized users.

## Introduction

Phenology provides excellent opportunities for studying climatic changes and their impacts on ecosystems. The changes in the annual cycle of plants are closely linked to the seasonal course of temperature, light and water supply, among other factors, and phenological observations constitute direct evidence of plants’ responses to these changes. With these regards, phenological data contribute tangibly to our understanding of global change biology (Menzel 2000, 2002; Parmesan and Yohe 2003; Piao et al. 2019; Menzel et al. 2020). In Europe, the plant phenological data have demonstrated an average advance of spring/summer 2.5 days per decade between the years 1971 and 2000, in accordance with instrumentally observed warming (Menzel et al. 2006). A more recent analysis of European phenological data (1971–2010) identified significant species-specific advancements in plant flowering onsets across the continent, with the highest magnitude of change 2.2 to 9.6 days per decade obtained for boreal conditions; moreover, more pronounced phenological changes were detected over the later decades, since 1991, in comparison with earlier years (Templ et al. 2017). While these trends demonstrate the value of phenological analyses in detecting the effects of recent and ongoing changes in growing season climate on ecosystems, they also indicate the instrumental role of high-latitude phenological observations, i.e. those from boreal and Arctic settings (Wielgolaski and Inouye 2013) in estimating the changes of presumably highest magnitude taking place over the most recent times.

Plant phenological data from boreal and subarctic settings are available from the Finnish National Phenological Network (FNPN), originally established by the Finnish Forest Research Institute in 1995 (Poikolainen et al. 1997; Kubin et al. 2006) to systematically follow the changes in forest ecological responses to climatic change (Kubin 1993). In Finland, plant phenological data have been continuously collected since 1750s with involvement of several institutional organisations and scientific societies 1750s (Kubin et al. 2008; Terhivuo et al. 2009; Holopainen et al. 2012). The more recent parts of the subsets of these data have previously been analysed mainly for purposes of the climatic analysis (Lappalainen and Heikinheimo 1992; Häkkinen et al. 1995; Holopainen et al. 2006, 2013; Linkosalo et al. 2009). Within the FNPN, the phenology of forest trees and shrubs was monitored since the spring of 1997 via a nationwide observation network in collaboration with universities, state research institutions, and vocational schools and colleges using a unified protocol (Kubin et al. 2007a, b). This collection of the data continued in cooperation with activities through the COST action 725 (Nekovář et al. 2008) with an objective of unifying the protocols of observations. Another objective was to create a common database of phenological observations through the continent, an undertaking now constituting the Pan European Phenological database PEP725 with updates of FNPN data (Koch et al. 2008; Templ et al. 2018). As of Jan 1, 2015, the Finnish Forest Research Institute was integrated into the Natural Resources Institute Finland, and the phenological observations were continued under this institute until the autumn of 2017.

Various parts of the FNPN dataset have been previously analysed. Pudas et al. (2008a) investigated the timing of *Betula pubescens* phenophases at 30 sites across Finland during 1997–2006. Pudas et al. (2008b) focussed on phenophases of various plant species observed only in the northernmost part of the country (Lapland) over the same period. Overall, they connected the advancing onsets of spring phenophases with year-to-year changes in the mean May temperature but found no trends in autumn phenophases (Pudas et al. 2008a, b). Terhivuo et al. (2009) presented an analysis of long-term phenological trends concentrating on *Prunus padus* and *Sorbus aucuparia* time-series, to place their recent flowering dates in the context of historical phenological data. They showed that the flowering dates had changed by 3 and 2 days later, as observed for *P. padus* and *S. aucuparia*, in comparison with the historical baseline. Ranta et al. (2010) used the leafing time-series of *Populus tremula* from thirty stations across the country (1997–2007) to analyse the synchrony in leafing as a function of the sites’ distances and temperature data in terms of the Moran effect to demonstrate that the phenological events can be synchronised the same way as population fluctuations. Poikolainen et al. (2016) analysed the trends in spring and autumn phenophases of *B. pubescens* observed at 21 sites (1997–2013) across the southern, middle, and northern boreal zones in Finland, in connection with annual temperature (in degree day units) cycle. During their study period, the bud burst had advanced 8 days and, in 2013, the average dates of 15, 18, and 26 May for southern, middle, and northern boreal sites were recorded. Moreover, the length of the growing period was, on average, 136, 122, and 130 days at the southern, middle, and northern boreal sites (Poikolainen et al. 2016).

Furthermore, the FNPN dataset is now part of the Pan European Phenological database PEP725 (Koch et al. 2008; Templ et al. 2018), the successor of the database developed through the COST action 725 (Nekovář et al. 2008), the PEP725 dataset currently consisting of more than ten million phenological time-series related to 121 wild plant and 144 cultivars from altogether ca. 20,000 different sites across Europe (Templ et al. 2018). Within this entity of data, Menzel et al. (2006) classified the FNPN data as among the national specialist networks. As a consequence, the FNPN data will be foreseeably investigated in a number of research projects and programs in the future. Recently, Templ et al. (2017) analysed the data of flowering dates of six plant species collected at 963 sites located between 40.9 and 67.9° N in twelve European countries (1970–2010), their analyses including the relevant parts of the FNPN dataset. Their results showed remarked advancements in plant flowering onsets, with a delay in this phenological stage with an increase in latitude, and an advance with changing climate (Templ et al. 2017).

The full FNPN dataset has not been hitherto described in the literature. The purpose of this study is to present these plant phenological observations from Finland covering the full period of data collection between the years 1997 and 2017. First, we itemise the dataset according to observed plant species, phenological stages, and phenophases (combining the species and its phenological stage). We also present the spatiotemporal coverage of the available observations. Second, the temporal trends in the phenological time-series (1997–2017) are assessed by their (slope) strengths, seasonal representativeness, and geographical variation. Third, a comparison is made where multiple within-site observations are available. Fourth, the results are discussed for the spring and autumn signals by highlighting their overall indications for climate research and characterising the FNPN data in the context of other relevant phenological data.

## Material and methods

### Plant phenological data

**Fig. 1:**
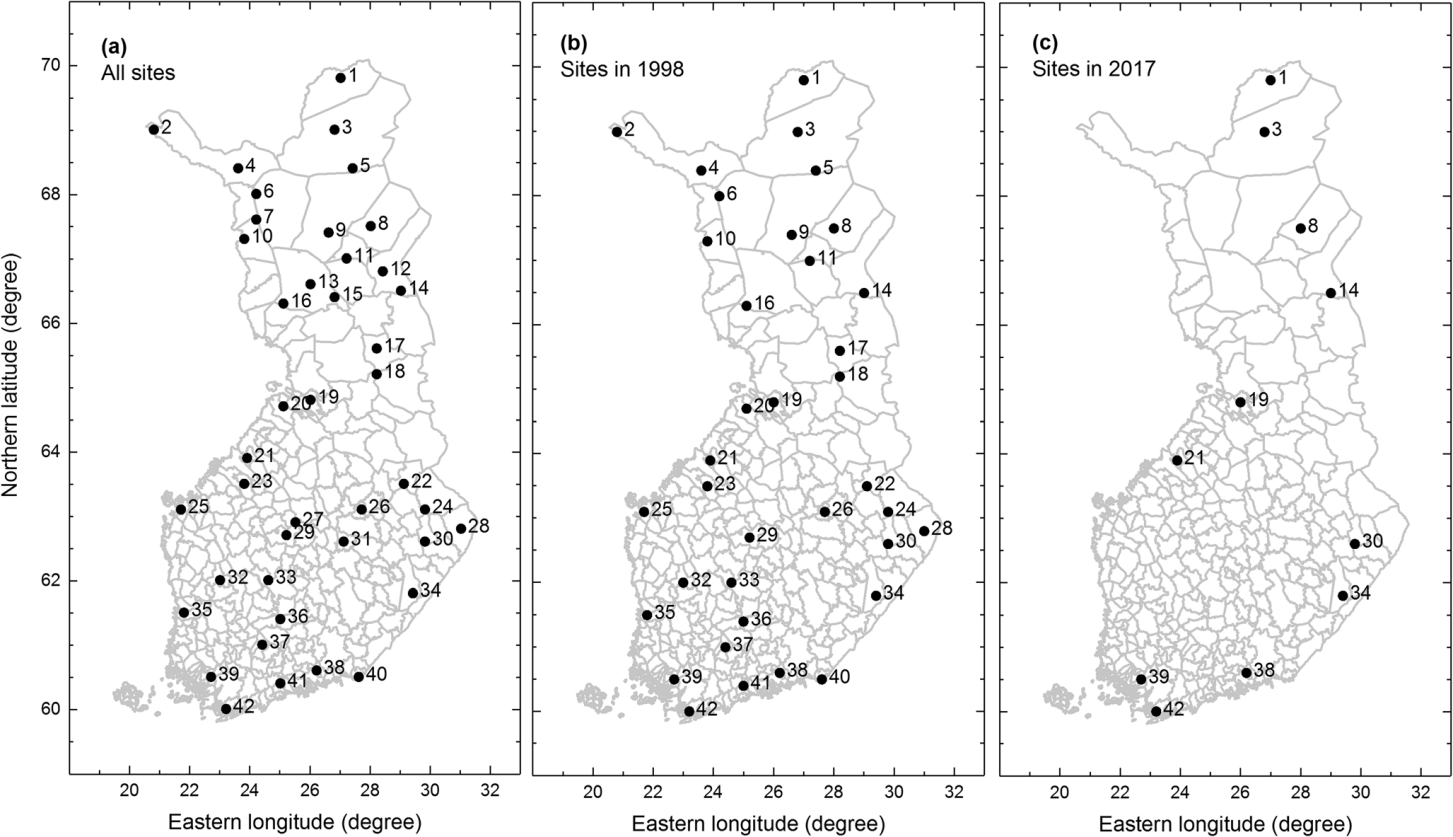
Maps of Finland with phenological observation sites over the full period (1750–1875) (**a**), and over the years of 1998 (**b**) and 2017 (**c**). The sites are numbered as follows: (1) Kevo, (2) Kilpisjärvi, (3) Muddusjärvi, (4) Hetta, (5) Saariselkä, (6) Pallasjärvi, (7) Äkäslompolo, (8) Värriö, (9) Sodankylä, (10) Kolari, (11) Pyhätunturi, (12) Salmivaara, (13) Apukka, (14) Oulanka, (15) Kivalo, (16) Pisavaara, (17) Taivalkoski, (18) Paljakka, (19) Muhos, (20) Ruukki, (21) Kannus, (22) Nurmes, (23) Veteli, (24) Koli, (25) Korsholm, (26) Siilinjärvi, (27) Pyhähäkki, (28) Mekrijärvi, (29) Kolkanlahti, (30) Joensuu, (31) Suonenjoki, (32) Parkano, (33) Vilppula, (34) Punkaharju, (35) Pori, (36) Vesijako, (37) Aulanko, (38) Lapinjärvi, (39) Preitilä, (40) Ravijoki, (41) Ruotsinkylä, and (42) Solböle

The dataset collected by the Finnish Forest Research Institute/Natural Resources Institute Finland contains altogether 43,589 plant phenological observations made by the stuff of the research institute using a ratified protocol (Kubin et al. 2007a, b) at 42 sites (between 70° N and 60° N, and 20° E and 30° E) (Fig. 1a) on eleven plant species typical to Finnish forests around the country between the years 1997 and 2017. No observations were made on the Åland Islands (an archipelago between the mainlands of Finland and Sweden). Phenological stages were recorded predominantly from five individual trees per site since 2007, after which ten additional trees were recorded per site since 2010 for improved representativeness of data. Medium-sized and healthy trees were included in these observations. The observations on shrubs were made within the monitoring squares (1 m^2^). Similar to change in observing the trees, their number was increased from one to five since 2007. These observations were made on fifteen phenophases: berries ripe, berries unripe, bud burst, bud scales opened, end of height growth, female flowering 100%, flowering, leaf colouring, leaf fall, leaves full-sized, leaves opened, onset of female flowering, onset of height growth, onset of male flowering, and shedding of seeds, in association with the BBCH codes (Meier 2001). The observed species were aspen (*Populus tremula* L.), bilberry (*Vaccinium myrtillus* L.), bird-cherry (*Prunus padus* L.), cowberry (*Vaccinium vitis-idaea* L.), downy birch (*Betula pubescens* Ehrh.), grey alder (*Alnus incana* (L.) Moench), common juniper (*Juniperus communis* L.), Norway spruce (*Picea abies* (L.) Karsten), rowan (*Sorbus aucuparia* L.), Scots pine (*Pinus sylvestris* L.), and silver birch (*Betula pendula* Roth). The abundance of flowering, berry yields, and seed crops as well as the observations on the most common damages affecting the trees was also monitored within the observation network. These data are not inherently phenological and were not included in this paper.

In this paper, the FNPN dataset was characterised for the observations per site, for phenological records (series of observations representing the species, phenological stage, and site), and time-series (records containing at least 10 observations over the 21-year study period).

### The vernal equinox

An important detail regarding the time-series of phenological observations relates to the mismatch between the length of the solar year and the slightly longer average year on the Gregorian calendar (Sagarin and Micheli 2001). The possibility of an overestimation of trends towards earlier spring signals, and the benefits of adopting the dates of phenological observations in relation to the beginning of astronomical spring (vernal equinox) rather than by calendar day, has been previously presented (Sagarin 2001, 2009). Here we follow these recommendations and report our phenological dates as the number of days elapsed since the vernal equinox, i.e. the date the sun crosses the celestial equator from the austral to the boreal hemisphere that have, over the years under this study, varied between 20 and 21 March.

### Characterising the data

The dataset was characterised by the distributions of the observations as a function of their seasonal, spatial, and temporal coverage, the number of species and phenological stages. Moreover, temporal trends in the phenological time-series were calculated using approaches commonly applied in analyses of time-series (e.g. Menzel 2000; Schaber and Badeck 2005; Terhivuo et al. 2009; Eastman et al. 2013; Hamunyela et al. 2013; Zhou et al. 2016; Williams et al. 2018). The Mann-Kendall (MK) statistic (Kendall 1970; Mann 1945) was used to determine the statistical significance of the trends, and the rate of change (slope) was estimated using the Theil-Sen approach, also known as Sen’s (1968) slope, with 95% confidence interval for the estimate (Conover 1980). These estimations were made using the mean phenological time-series produced by averaging the *n* = 1–15 observations available on same phenophase (combining the species and phenological stage) for each site. Slope estimates were also compared with the sites’ latitudes and longitudes using the Spearman correlation coefficient (Conover 1980).

### Data reproducibility

Observations were made on multiple trees/monitoring squares since 2007. When more than single tree/monitoring square observations were available, the within-site observations for the same phenophase were averaged to compare between the dates of single and mean observations. First, the onset dates were compared between the dates of single and mean observations. The differences between the dates were calculated and classified according to the phenological stage. Second, the rates of change in the phenological time-series were estimated, separately for the series based on single and mean observations, using the Sen’s (1968) slope. Potential changes in the rate of change (days decade^−1^) as well as in the statistical significance of the trends, based on the MK-statistic (Kendall, 1970; Mann, 1945), were compared for information on data reproducibility.

## Results

### Characterising the dataset

#### Spatiotemporal distribution

**Fig. 2:**
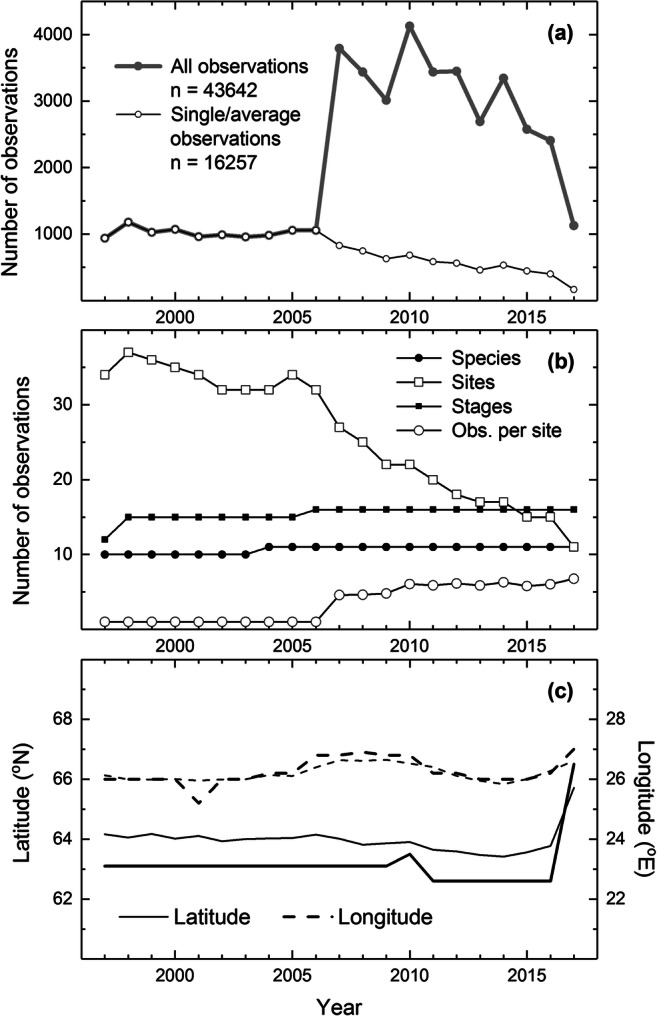
Temporal variations in the numbers of plant phenological observations given as the number of all observations and those on single or averaged data from each site (**a**), and as the numbers of observed species, sites and phenological stages, the (mean) number of replications per site (**b**), and mean (grey lines) and median (black lines) latitudes and longitudes for *n* = 16,257 observations (**c**)

Over the first 10 years of the study period, the number of phenological observations was approximately one thousand per year. Since that date, the change in the monitoring protocol markedly increased the total number of observations (Fig. 2a). That is, there was an increase in the number of trees and monitoring squares (in the case of shrubs) observed per site, i.e. in the number of within-site observations since 2007 (Fig. 2b).

Averaging the within-site observations for the same phenophase allows for a more reasonable comparison between the early (1997–2006) and late (2007–2017) observation periods. This averaging of dates (rounded to the closest integer) reduces the number of available data points to *n* = 16,257 observations (this data is used hereafter for characterising the dataset, except for comparisons between single and mean dates). The number of mean observations (black line with open circles in Fig. 2a) shows that there was a steady decline in the number of observations over the late period. This decline is explained by the reducing number of observation sites over the same period (Fig. 2b). Comparison between the years 1998 and 2017 exemplifies the change in the spatial distribution of the sites for years with the highest (Fig. 1b) and lowest (Fig. 1c) number of sites. This shows that there are increasing gaps especially in the west-central Finland and northwest Finland (western Finnish Lapland) towards the end of the study period. By contrast, the numbers of observed plant species and phenological stages remained more or less constant (Fig. 2b). Since 2004, the observations were made on grey alder, an arboreal species of which phenology was not observed in this network before that date.

The coordinates of the 42 sites averaged 64.5° N and 25.6° E. Time-dependent summary statistics for *n* = 16,257 observations showed no marked fluctuations, an exception for the (last) year 2017 during which the observations represented latitudes around 66° N (Fig. 2c). Overall, this long-term stability would suggest that the geographical distribution of the observation sites was not subject to latitudinal or longitudinal shifts that would represent any large deviations from the central tendency.

#### Phenophases and time-series

The five most commonly observed species were Scots pine, downy birch, rowan, silver birch, and Norway spruce. Their observations accounted for 64.9% of the total amount, whereas the most commonly observed types of phenological stages were associated with the flowering, male flowering, berries ripe, leaves full-sized, and leaf colouring, and these observations accounting for 49.7% of the total amount.

There were altogether 40 combinations of species and their phenological stages, i.e. phenophases. The five most commonly observed phenophases were the budburst of downy birch, the flowering of bilberry, the downy birch leaves full-sized, the onset of height growth of Scots pine, and the bilberry berries unripe. These observations accounted for 16.0% of the total amount. Fifty percent and 90% of the observations were covered by 17 ad 33 most commonly observed phenophases.

**Fig. 3:**
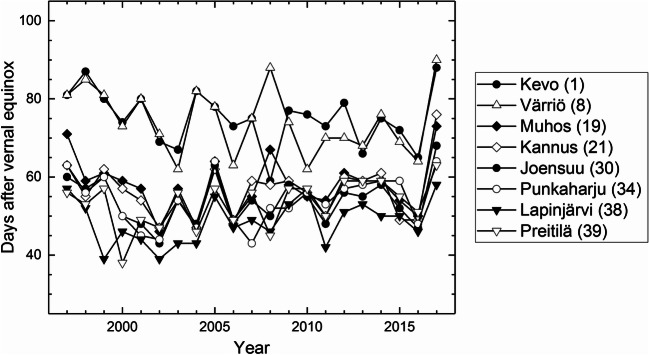
Phenological time-series of the bud burst of downy birch observed at eight sites, representing the latitudes between 70° N and 60° N, through the 21-year period (1997–2017) (for site names, see Fig. 1a)

There were altogether *n* = 1524 phenological records representing the species, phenological stage, and site. As many as 66 of these records contained 21 observations, that is, they cover the full study period (1997–2017). The most frequent of them is the bud burst of downy birch, observed continuously over the 21-year study period at eight sites (Fig. 3). Moreover, there are four other 21-year time-series observed at least at five sites: the downy birch leaves full-sized, the leaf colouring of downy birch, the bud burst of silver birch, and the flowering of bilberry. In addition to these 21-year time-series, there are one data point missing from 122 time-series, 85 time-series with two data points missing, and 83 time-series with three missing data points.

More than half of the phenological records (*n* = 808) contained at least 10 observations. These time-series contain a total number of *n* = 12,862 observations representing 39 phenophases. This dataset was used for detecting the temporal trends in the phenological time-series in the following sections, if not otherwise indicated.

### Temporal trends

#### Assessment of temporal trends

The *n* = 808 phenological records with at least 10 years of data were used for trend analyses. The minimum length of 10 years of data was chosen to perform the analyses of trends (Table S1) for a sizeable amount of the records. Sixty-two percent (*n* = 500) of the time-series exhibited negative trends (Sen’s slope), of which 20% (*n* = 99) were statistically significant (*p* < 0.05). At the same time, 30% (*n* = 244) of the time-series showed positive trends, of which 14% (*n* = 35) were statistically significant (*p* < 0.05). Eight percent (*n* = 64) of the time-series showed zero slope.

**Fig. 4:**
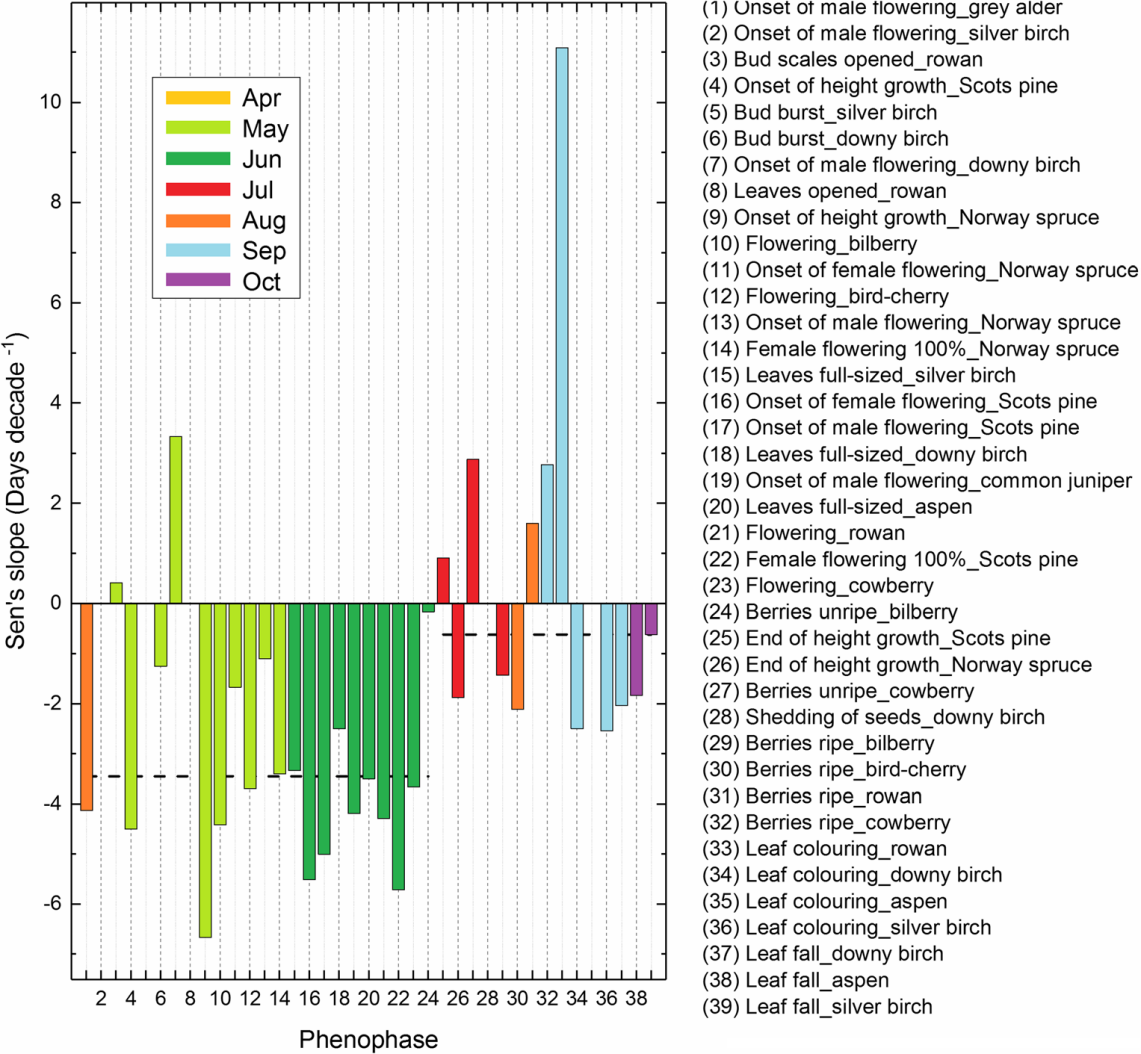
Trends in time-series of phenological observations over the 21-year period (1997–2017). Vertical axis indicates the median of the slopes estimated for each of the phenophases. Horizontal dashed lines represent the median levels of the spring and early-summer (April–June) and summer/autumn (July–October) phenophases. For actual values, see Table S1

Furthermore, the slopes were grouped according to the phenophase (Fig. 4). Such an examination revealed that the temporal trends were excessively negative for spring (April–May) and early-summer (June) phenology, a result signalling earlier onset of the growing season. The trend slopes for these (April–June) phenophases were negative in 23 out of 24 cases, their median value indicating an overall change of 3.4 days decade^−1^. Positive trends were observed for a limited number of summer (July–August) and autumn (September) phenophases which would provide an indication of delayed phenology in six out of 15 cases. However, the median value for these slopes (for phenophases occurring in July through October) remained negative with an indication of an overall change of 0.6 days decade^−1^. That is, the phenological indications of the later part of the growing season were clearly more variable with markedly less consistent than those observed for spring and early-summer phenology.

#### Geographical variation in trends

Comparisons of the trend strengths and sites’ coordinates indicated that there might be some weak latitude- and longitude-dependent variations. Negative and statistically significant (*p* < 0.001) correlation was found between the slope estimates and sites’ latitudes for spring and early-summer phenophases (Table 1), indicating relatively stronger signals of advancing spring and early-summer phenology towards the northern part of the country. Moreover, negative and statistically significant (*p* < 0.05) correlation was also obtained between the slope estimates and sites’ longitudes for spring and early-summer phenology, implying somewhat a stronger signal of advancing spring towards the eastern part of the country. For summer/autumn phenophases, by contrast, the trend slope estimates and sites’ longitudes were positively and statistically significantly (*p* < 0.05) correlated, indicating progressively later summer/autumn phenology towards the eastern part of the country. That the mean location of the observations sites did not illustrate considerable shifts through time (Fig. 2c) suggests no corresponding biases in geographical variations in the trends as observed here.

**Table 1:** Correlations between the trend slopes for the phenological time-series and their sites’ latitudes and longitudes, and the number of phenological time-series (*n*) having at least ten data points over the 21-year period (1997–2017). Calculations were done for the groups of phenological data representing spring/early-summer (April–June; AMJ) and summer/autumn (July–October; JASO) trends (see Fig. 4). Statistical significance at levels *p* < 0.05, *p* < 0.01, and *p* < 0.001 are denoted by one (*), two (**), and three (***) asterisks, respectively

	Latitude	Longitude	*n*
AMJ	− 0.284***	− 0.100*	513
JASO	0.043	0.119*	295

### Single and mean dates

**Fig. 5:**
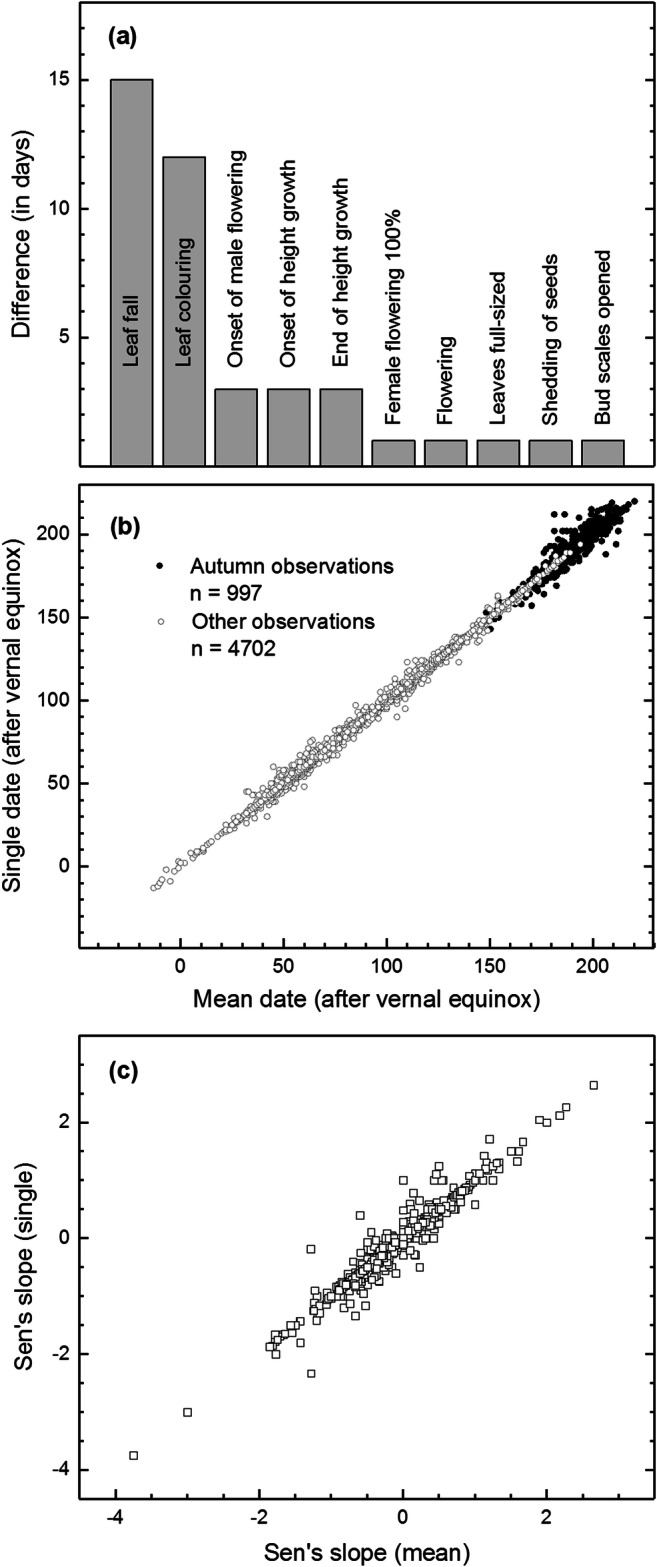
Comparison between the single and mean observations per site. Phenological stages with sizeable difference (≥ 10 days) between the dates of single and mean observations (**a**). Comparison between the autumn (leaf fall and leaf colouring) and other observations based on single and mean dates (**b**). Comparison of the *n* = 798 Sen’s slope (days year^−1^) estimates obtained for the phenological time-series based on single and mean observations over the 21-year period (1997–2017) (**c**)

Altogether, *n* = 5699 observations were based on more than single tree/monitoring square, making it possible to compare between the dates of single and mean observations. The majority of these observations (62.8%) were identical, that is, the dates based on single tree/monitoring square (remaining the same through the study period) were exactly the same as those based on observations on multiple trees/monitoring squares. An additional one-third (32.1%) of observations had a difference less than 5 days. The remaining observations with larger within-site difference were clearly dominated by autumn phenology (Fig. 5a, b). Phenological stages such as leaf colouring and leaf fall together accounted for approximately half (53.7%) and two-thirds (65.9%) of the dates with at least 5 and 10 days difference, respectively.

Focussing on the phenological time-series based one tree/monitoring square reduced the amount of phenological data to *n* = 798 time-series (with at least ten data points over the 21-year period). Comparison between the trends assessed for the two types of time-series showed no large deviations (Fig. 5c). Nearly half of the time-series (47.5%) showed a difference in temporal trends of less than 0.1 days decade^−1^, judged from their trend estimates, and more than three quarters of them (77.1%) less than 1 day decade^−1^. Again, most of the changes in the significance levels of the trends were observed for leaf colouring and leaf fall. Nine out of sixteen time-series of leaf colouring and leaf fall were non-significant for the time-series based on mean dates but significant (*p* < 0.05) for dates based on one tree/monitoring square. Clearly, the results suggest that multitude of observations would warrant more unbiased estimates for these observations of autumn phenology (i.e. leaf colouring and leaf fall).

## Discussion

### Spring signals

We observed a clear signal of advancing spring and early-summer phenology over the 1997–2017 period. These indications concur with several previous phenological findings on national and European level. An average advance of spring/summer 2.5 days decade^−1^ in Europe between the years 1971 and 2000 (Menzel et al. 2006) was more recently exceeded by estimations of flowering onsets advancing 2.2–9.6 days decade^−1^ as specifically obtained for boreal conditions over an extended period (1971–2010) (Templ et al. 2017). Calculated from data deposited in the PEP725 database, where the observations north of 60° N are excessively of Finnish origin (see Templ et al. 2018), i.e. the FNPN dataset, the latter trend estimates ought to agree particularly with our results. The average advance of spring/early-summer phenology obtained in this study as 3.4 days decade^−1^ (Fig. 4) is rather compatible with both of the foregoing European-scale estimates, more so with those of Templ et al. (2017), this agreeing also with their remark that more pronounced phenological changes are to be found in the boreal conditions, i.e. northern Europe and over the recent years.

Comparisons with previous FNPN studies, however, show that the average advance of spring/early-summer phenology did not quite reach those derived specifically for the spring phenophases in northern part of the country (10–20 days decade^−1^) (Pudas et al. 2008b) or for the bud burst of *Betula pubescens* through the country during the 1997–2006 period (7–14 days decade^−1^) (Pudas et al. 2008a). More recently, however, the timing of bud burst of *B. pubescens* was found to advance by 5 days decade^−1^ in the northern boreal zone (i.e. northern Finland) but to remain constant in the southern and middle boreal zones in Finland; these estimations employing the FNPN data from 1997 to 2013 (Poikolainen et al. 2016). These differences could be explained by at least two possible factors. First, the shorter period of investigation (Pudas et al. 2008a, b) may have resulted in steeper slopes estimates. This reasoning would resonate with that of Poikolainen et al. (2016) who noticed the potential biases in estimating trend for short phenological time-series. Second, the stronger trends in the northern part of the country (Pudas et al. 2008b) may be simply explained by our results showing a latitudinal correlation indicative of more prominent signals of advancing spring and early-summer phenology towards the north (Table 1).

More generally, the signals of spring advance have been reported in Finnish phenological studies for trees, shrubs, grasses, agricultural plants, migratory birds, and insects (Lappalainen et al. 2008; Linkosalo et al. 2009; Holopainen et al. 2013) as well as for lake and river ice breakups (Helama et al. 2013; Korhonen 2019; Norrgård and Helama 2019). Obtained from data representative of plant and animal kingdoms, biotic and abiotic phenomena and over various time intervals, these studies add to the accumulating evidence suggesting various types of natural systems being largely driven by a common climatic forcing. Long-term warming of spring temperatures has in fact been shown to have continued in Finland over the past 100–150 years (Tuomenvirta 2004; Tietäväinen et al. 2010), this trend of warming remaining verified also by palaeoclimatic evidence (Holopainen et al. 2009).

### Autumn signals

The autumn signal was, in general, much less consistent than that observed for the advancing spring. First, the proportion of positive trends increased towards the later part of the growing season whereas the overall level of the trend slopes remained slightly negative for phenophases occurring in July through October (see Fig. 4). Second, the autumn phenology (pertaining at least to leaf colouring and leaf fall) was more variable within each site which inevitably results in some loss of accuracy (Fig. 5a, b). Even so, the phenology based on mean dates did correspond with that based on single observations, which lent support to initial indications also of autumn phenology. Even so, it remains obvious that observations of at least leaf colouring and leaf fall show more within-site variations than the other phenological stages (Fig. 5a) and that multitude of observations per site would warrant more widely reliable estimates of these autumnal stages.

That the spring and early-summer phenology is advancing and some of the phenophases occurring in summer/autumn delaying could potentially indicate that the growing season is lengthening. Interestingly, this would also agree with calculations and projections showing prolongations of thermal growing season in the same region over the past and future decades (Linderholm et al. 2008; Ruosteenoja et al. 2011). It is notable, however, that the FNPN data shows also a number of observations indicative of progressively earlier summer/autumn phenology (see Fig. 4). This inconsistency demonstrates that there is perhaps no assured way to reasonably interpret the finer details of this potential change from our results. Likely, it can be interpreted as being due to noise whose intensity exceeds that present in spring and early-summer phenology. This line of reasoning would generally follow the results showing more ambiguous trends for the autumn phenology, in comparison with spring phenology, at least in boreal conditions (Pudas et al. 2008a, b; Poikolainen et al. 2016). Intriguingly, the leaf colouring of birch in northern Finland, included in a previous climatic analysis of Holopainen et al. (2013), was found to be best explained by spring (May) rather than autumn temperatures, albeit it could be emphasised that temperature correlations with this phenological stage were generally low. Results with similar implications were reported from a European-scale study finding no clear relationship between trends of temperature and leaf colouring/leaf fall (Menzel et al. 2006). These findings would not be at odds with recent indications suggesting that autumn phenology can be predicted using spring phenology (Keenan and Richardson 2015).

### The FNPN/Finnish phenological data

The FNPN dataset is not the only assemblage of phenological data in Finland. Historically, the importance of collecting phenological data was advocated by Carl von Linné who also initiated the station network (Dahl and Langvall 2008). Soon after, however, as pointed out by Holopainen et al. (2018), Linné’s recommendation to create phenological datasets was remembered only in Finland where the efforts have indeed continued by several generations of volunteers, with varying levels of institutional enthusiasm to organise the activity, since 1750s. Indeed, several institutional organisations and scientific societies have been involved in this long-lasting process, the history of which itself has been reviewed in previous publications (Kubin et al. 2008; Terhivuo et al. 2009; Holopainen et al. 2012). A large body of this work until the 1870s was compiled and published already by Moberg (1857, 1860, 1885, 1894) from whose works the plant data has been recently digitised (Holopainen et al. 2018), the more recent parts of the subsets of the corresponding data being digitised and analysed mainly for purposes of the climatic analysis (Lappalainen and Heikinheimo 1992; Häkkinen et al. 1995; Holopainen et al. 2006, 2013; Linkosalo et al. 2009). Compared with these data, the FNPN dataset may form the most consistent and homogenous collection of observations. In the context of European phenological data, it could be classified among the national specialist networks (Menzel et al. 2006), in comparison with the volunteering activities.

The FNPN dataset consists of observations made on eleven tree and shrubs species and fifteen phenological stages. These data constitute forty different variables of corresponding phenophases collected using a unified protocol (Kubin et al. 2007a, b) at 42 sites between 70° N and 60° N. As such, the FNPN data provides a strong high-latitude contribution to the European Phenological database PEP725 (Koch et al. 2008; Templ et al. 2018) over the 21-year period of available observations. That is, the recently published version of the PEP725 database (Templ et al. 2018) contains almost no sites north of 60° N other than FNPN observations and is entirely based on FNPN data north of around 65° N. Yet, the boreal phenology was recently shown to exhibit significant advancements in plant flowering onset dates with magnitudes exceeding the less pronounced changes in other biogeographical regions (continental, alpine, Pannonian, and Mediterranean) (Templ et al. 2017); these results demonstrating the importance of understanding the high-latitude phenological responses to climatic changes.

This study provides a characterisation of this northern dataset with outlines of assessing the trends in the FNPN-based phenological time-series over the 1997–2017 period. Even so, the spring and summer seasons of 2018 were marked by unusually hot weather across northern and central Europe and, according to Albergel et al. (2019), the summer 2018 was warmest in Europe since continental records began in 1910. In Finland, May and July 2018 were exceptionally warm, the July temperature anomalies reaching their extremes in northern Finland (Sinclair et al. 2019). Moreover, Vogel et al. (2019) attributed the heat extremes experienced during the same summer across the northern hemisphere to human-induced climate change and Albergel et al. (2019) showed the impacts of this heatwave on leaf area index of which negative anomalies exceeded two standard deviations from the mean in north-western Europe both for June through October season and for July. Cessation of phenological observations in 2017 due to decline in financial support precludes any investigations of the potential effects from this anomalous heat on phenology in the context of the FNPN dataset. Ironically, the situation demonstrates the continuous need for data collection to provide evidence relating to advancing climatic changes and anomalies.

## Electronic supplementary material

ESM 1 (PDF 60 kb)
