# Supplementary material for: Finnish National Phenological Network 1997–2017: from observations to trend detection
Source: Int J Biometeorol. 2020 Jul 6;64(10):1783–93. doi: 10.1007/s00484-020-01961-6 (PMC7481168; doi:10.1007/s00484-020-01961-6)
Supplement: Supplementary file 1 — (PDF 60 kb) [file 484_2020_1961_MOESM1_ESM.pdf]

Supplementary Information

# Finnish National Phenological Network 1997−2017: from observations to trend detection

Helama S, Tolvanen A, Karhu J, Poikolainen J, Kubin E

Content:

- Table S1. Trends in phenological records

Table S1. Trends in phenological records. Plant species were referred to by their EPPO codes (EPPO code system, http://eppt.eppo.org/) and the phenological stage by their BBCH codes (Kubin et al. 2007a, 2007b). Site numbers refer to Fig. 1 in the main text. The trends were quantified using the Mann-Kendall statistic (MK) and its statistical significance (p), the Sen's slope (S; days year$^{-1}$) and its 95% confidence interval ($S^r$, $S^s$), and the number of years (n) over which the statistics were computed.

| Species | Phase | Site | MK | p | S | Sr | Ss | n |
|---|---|---|---|---|---|---|---|---|
| ALUIN | BBCH61 | 1 | 0.4 | ns | 0.25 | -2.17 | 1.5 | 11 |
| ALUIN | BBCH61 | 14 | -0.4 | ns | -0.33 | -1.67 | 1 | 11 |
| ALUIN | BBCH61 | 19 | 0.5 | ns | 0.31 | -2.25 | 1.5 | 12 |
| ALUIN | BBCH61 | 21 | -0.7 | ns | -0.52 | -3.14 | 0.86 | 12 |
| ALUIN | BBCH61 | 30 | -0.4 | ns | -0.45 | -2 | 0.86 | 13 |
| ALUIN | BBCH61 | 34 | -0.6 | ns | -0.38 | -3.6 | 1.25 | 11 |
| ALUIN | BBCH61 | 38 | -1.3 | ns | -1 | -4 | 1.67 | 10 |
| ALUIN | BBCH61 | 39 | -1.3 | ns | -1.55 | -4.33 | 1.13 | 14 |
| BETPB | BBCH07 | 1 | -1.7 | ns | -0.38 | -0.92 | 0.14 | 21 |
| BETPB | BBCH07 | 2 | -1.6 | ns | -0.75 | -2.33 | 0.43 | 11 |
| BETPB | BBCH07 | 3 | -0.6 | ns | -0.21 | -0.87 | 0.5 | 20 |
| BETPB | BBCH07 | 5 | -0.8 | ns | -1.17 | -3 | 1 | 10 |
| BETPB | BBCH07 | 6 | -2.1 | <0.05 | -1.2 | -3.44 | -0.25 | 14 |
| BETPB | BBCH07 | 8 | -1.6 | ns | -0.57 | -1.11 | 0.21 | 21 |
| BETPB | BBCH07 | 10 | -0.6 | ns | -0.18 | -0.72 | 0.57 | 20 |
| BETPB | BBCH07 | 14 | 0.5 | ns | 0.1 | -0.64 | 0.89 | 19 |
| BETPB | BBCH07 | 18 | 0.1 | ns | 0 | -1 | 0.83 | 18 |
| BETPB | BBCH07 | 19 | -0.5 | ns | -0.13 | -0.67 | 0.67 | 21 |
| BETPB | BBCH07 | 21 | 0.1 | ns | 0 | -0.5 | 0.5 | 21 |
| BETPB | BBCH07 | 22 | -1.2 | ns | -0.4 | -1.4 | 0.63 | 15 |
| BETPB | BBCH07 | 24 | -0.1 | ns | -0.16 | -1.86 | 1.75 | 13 |
| BETPB | BBCH07 | 26 | -1.2 | ns | -1 | -3.5 | 1 | 11 |
| BETPB | BBCH07 | 28 | 0.4 | ns | 0.44 | -1.25 | 1 | 14 |
| BETPB | BBCH07 | 29 | -0.9 | ns | -0.83 | -2.5 | 1.4 | 10 |
| BETPB | BBCH07 | 30 | 0.1 | ns | 0 | -0.6 | 0.73 | 21 |
| BETPB | BBCH07 | 31 | -0.6 | ns | -0.11 | -0.83 | 0.8 | 17 |
| BETPB | BBCH07 | 32 | 1.9 | ns | 0.33 | -0.08 | 0.92 | 19 |
| BETPB | BBCH07 | 34 | 1.1 | ns | 0.41 | -0.33 | 1 | 21 |
| BETPB | BBCH07 | 36 | -0.5 | ns | -0.13 | -0.86 | 0.5 | 20 |
| BETPB | BBCH07 | 37 | -0.1 | ns | -0.16 | -1.5 | 0.83 | 12 |
| BETPB | BBCH07 | 38 | 1.3 | ns | 0.35 | -0.33 | 0.88 | 21 |
| BETPB | BBCH07 | 39 | 2 | ns | 0.33 | 0.08 | 0.88 | 21 |
| BETPB | BBCH07 | 40 | 0.4 | ns | 0 | -1 | 1.67 | 11 |
| BETPB | BBCH07 | 41 | -0.2 | ns | 0 | -0.6 | 0.5 | 19 |
| BETPB | BBCH07 | 42 | 2.2 | <0.05 | 0.52 | 0.13 | 1.11 | 20 |
| BETPB | BBCH15 | 1 | -2.4 | <0.05 | -0.9 | -1.75 | -0.22 | 20 |
| BETPB | BBCH15 | 2 | -0.6 | ns | -0.3 | -1.8 | 1 | 11 |
| BETPB | BBCH15 | 3 | -0.8 | ns | -0.19 | -0.86 | 0.5 | 21 |

| | | | | | | | | |
|---|---|---|---|---|---|---|---|---|
| BETPB | BBCH15 | 5 | -0.6 | ns | -0.44 | -3.4 | 1.25 | 10 |
| BETPB | BBCH15 | 6 | -0.3 | ns | -0.12 | -0.88 | 1 | 12 |
| BETPB | BBCH15 | 8 | 2.1 | <0.05 | 0.79 | 0.1 | 1.3 | 21 |
| BETPB | BBCH15 | 10 | -2.2 | <0.05 | -0.95 | -1.89 | -0.29 | 20 |
| BETPB | BBCH15 | 14 | -0.6 | ns | -0.2 | -1.2 | 0.83 | 19 |
| BETPB | BBCH15 | 18 | -1.8 | ns | -0.5 | -1.25 | 0.13 | 18 |
| BETPB | BBCH15 | 19 | -0.9 | ns | -0.25 | -0.71 | 0.4 | 21 |
| BETPB | BBCH15 | 21 | -1.1 | ns | -0.5 | -1.35 | 0.43 | 21 |
| BETPB | BBCH15 | 22 | -1.1 | ns | -0.4 | -1.5 | 0.36 | 15 |
| BETPB | BBCH15 | 24 | 1.1 | ns | 0.33 | -0.8 | 1.6 | 13 |
| BETPB | BBCH15 | 26 | -2 | ns | -1.5 | -2.67 | -0.14 | 11 |
| BETPB | BBCH15 | 28 | -1 | ns | -0.44 | -1.8 | 0.4 | 12 |
| BETPB | BBCH15 | 29 | -2.7 | <0.05 | -1.78 | -3 | -1 | 10 |
| BETPB | BBCH15 | 30 | -0.8 | ns | -0.15 | -0.79 | 0.4 | 20 |
| BETPB | BBCH15 | 31 | -2.5 | <0.05 | -1.04 | -2 | -0.38 | 17 |
| BETPB | BBCH15 | 32 | -0.5 | ns | -0.13 | -0.92 | 0.5 | 17 |
| BETPB | BBCH15 | 34 | -2 | ns | -0.52 | -1.25 | -0.07 | 20 |
| BETPB | BBCH15 | 36 | -1.7 | ns | -0.67 | -1.5 | 0.29 | 19 |
| BETPB | BBCH15 | 37 | 0.8 | ns | 0.42 | -1.33 | 2.77 | 12 |
| BETPB | BBCH15 | 38 | 3 | <0.01 | 1 | 0.5 | 1.5 | 21 |
| BETPB | BBCH15 | 39 | 1.7 | ns | 0.33 | -0.13 | 0.83 | 20 |
| BETPB | BBCH15 | 40 | 0 | ns | 0 | -1.8 | 1.4 | 11 |
| BETPB | BBCH15 | 41 | 1.6 | ns | 0.48 | -0.25 | 1.2 | 17 |
| BETPB | BBCH15 | 42 | 1.2 | ns | 0.41 | -0.33 | 1 | 17 |
| BETPB | BBCH61 | 1 | 0.6 | ns | 0.56 | -1.8 | 2.86 | 12 |
| BETPB | BBCH61 | 19 | 0 | ns | 0 | -2 | 2.33 | 11 |
| BETPB | BBCH61 | 34 | 1.4 | ns | 0.4 | -0.44 | 1 | 10 |
| BETPB | BBCH61 | 38 | -0.2 | ns | 0 | -1 | 1 | 11 |
| BETPB | BBCH61 | 39 | 0.3 | ns | 0.33 | -0.5 | 1.67 | 11 |
| BETPB | BBCH89 | 38 | 0.3 | ns | 0 | -0.67 | 2 | 12 |
| BETPB | BBCH92 | 1 | -1.2 | ns | -0.25 | -0.8 | 0.21 | 19 |
| BETPB | BBCH92 | 2 | 0.6 | ns | 0.43 | -2 | 3 | 11 |
| BETPB | BBCH92 | 3 | -1.8 | ns | -0.42 | -0.9 | 0.1 | 21 |
| BETPB | BBCH92 | 5 | 0.4 | ns | 0.33 | -1.43 | 1 | 10 |
| BETPB | BBCH92 | 6 | -1 | ns | -0.22 | -1 | 0.5 | 14 |
| BETPB | BBCH92 | 8 | -2.3 | <0.05 | -0.4 | -1 | -0.13 | 20 |
| BETPB | BBCH92 | 10 | -2.6 | <0.05 | -0.92 | -1.57 | -0.38 | 18 |
| BETPB | BBCH92 | 14 | -1.2 | ns | -0.2 | -0.71 | 0.23 | 18 |
| BETPB | BBCH92 | 18 | -1.1 | ns | -0.25 | -0.67 | 0.46 | 18 |
| BETPB | BBCH92 | 19 | -0.5 | ns | -0.15 | -0.75 | 0.5 | 21 |
| BETPB | BBCH92 | 21 | -1.5 | ns | -0.5 | -1.33 | 0.25 | 21 |
| BETPB | BBCH92 | 22 | 0.3 | ns | 0 | -1 | 1 | 15 |
| BETPB | BBCH92 | 24 | -0.1 | ns | 0 | -0.75 | 0.8 | 13 |
| BETPB | BBCH92 | 30 | 3 | <0.01 | 0.58 | 0.27 | 1.13 | 20 |
| BETPB | BBCH92 | 32 | -2 | ns | -0.67 | -1.67 | -0.11 | 15 |
| BETPB | BBCH92 | 34 | -1.3 | ns | -0.25 | -0.58 | 0.24 | 21 |
| BETPB | BBCH92 | 36 | -2 | ns | -0.37 | -0.86 | -0.06 | 16 |
| BETPB | BBCH92 | 37 | -0.9 | ns | -0.46 | -2.57 | 1 | 13 |

| | | | | | | | | |
|---|---|---|---|---|---|---|---|---|
| BETPB | BBCH92 | 38 | 0.6 | ns | 0.16 | -0.5 | 0.8 | 20 |
| BETPB | BBCH92 | 39 | -0.3 | ns | 0 | -0.44 | 0.4 | 21 |
| BETPB | BBCH92 | 41 | -1.9 | ns | -0.5 | -1 | 0.13 | 17 |
| BETPB | BBCH92 | 42 | -2 | ns | -0.6 | -1.22 | -0.15 | 18 |
| BETPB | BBCH97 | 1 | -1.4 | ns | -0.39 | -1 | 0.33 | 20 |
| BETPB | BBCH97 | 2 | 0.5 | ns | 0.57 | -1.33 | 1.5 | 11 |
| BETPB | BBCH97 | 3 | -0.5 | ns | -0.21 | -0.92 | 1 | 20 |
| BETPB | BBCH97 | 5 | 0.1 | ns | 0.5 | -2 | 1.86 | 10 |
| BETPB | BBCH97 | 6 | -1.4 | ns | -0.58 | -1.67 | 0.5 | 14 |
| BETPB | BBCH97 | 8 | -2.7 | <0.05 | -0.67 | -1.38 | -0.29 | 19 |
| BETPB | BBCH97 | 10 | -0.2 | ns | -0.19 | -1 | 0.9 | 17 |
| BETPB | BBCH97 | 14 | -1.6 | ns | -0.22 | -0.67 | 0.11 | 18 |
| BETPB | BBCH97 | 18 | 0.6 | ns | 0.29 | -0.63 | 1.22 | 16 |
| BETPB | BBCH97 | 19 | -1.2 | ns | -0.14 | -0.5 | 0.27 | 21 |
| BETPB | BBCH97 | 21 | -2.4 | <0.05 | -1 | -2 | -0.29 | 20 |
| BETPB | BBCH97 | 22 | -0.4 | ns | -0.2 | -1.17 | 0.67 | 13 |
| BETPB | BBCH97 | 24 | -0.5 | ns | -0.24 | -1.29 | 1 | 13 |
| BETPB | BBCH97 | 30 | 0.5 | ns | 0.09 | -0.5 | 0.69 | 20 |
| BETPB | BBCH97 | 32 | -1.6 | ns | -0.7 | -1.4 | 0.2 | 18 |
| BETPB | BBCH97 | 34 | -0.7 | ns | -0.19 | -0.64 | 0.38 | 20 |
| BETPB | BBCH97 | 36 | 0 | ns | 0 | -0.67 | 0.7 | 15 |
| BETPB | BBCH97 | 37 | 0.8 | ns | 0.5 | -2.33 | 2.83 | 12 |
| BETPB | BBCH97 | 38 | 1.3 | ns | 0.36 | -0.33 | 0.8 | 20 |
| BETPB | BBCH97 | 39 | -1.2 | ns | -0.25 | -1 | 0.35 | 21 |
| BETPB | BBCH97 | 41 | -1.4 | ns | -0.33 | -1 | 0.5 | 17 |
| BETPB | BBCH97 | 42 | -3.2 | <0.01 | -1.29 | -2.2 | -0.67 | 14 |
| BETPE | BBCH07 | 5 | -0.8 | ns | -1.17 | -3 | 1 | 10 |
| BETPE | BBCH07 | 8 | -1.6 | ns | -0.56 | -1.07 | 0.14 | 21 |
| BETPE | BBCH07 | 10 | -0.6 | ns | -0.16 | -0.67 | 0.5 | 20 |
| BETPE | BBCH07 | 14 | 0.5 | ns | 0.19 | -0.5 | 0.83 | 19 |
| BETPE | BBCH07 | 18 | 0 | ns | 0 | -1 | 0.67 | 18 |
| BETPE | BBCH07 | 19 | -0.4 | ns | -0.09 | -0.75 | 0.5 | 21 |
| BETPE | BBCH07 | 21 | -0.5 | ns | -0.18 | -0.78 | 0.43 | 20 |
| BETPE | BBCH07 | 22 | -1.5 | ns | -0.6 | -1.83 | 0.43 | 15 |
| BETPE | BBCH07 | 24 | -0.5 | ns | -0.53 | -2.33 | 1.33 | 13 |
| BETPE | BBCH07 | 26 | -0.8 | ns | -0.5 | -2 | 1 | 11 |
| BETPE | BBCH07 | 28 | -0.4 | ns | -0.27 | -1.6 | 1.11 | 14 |
| BETPE | BBCH07 | 29 | -1 | ns | -1 | -3 | 1.33 | 10 |
| BETPE | BBCH07 | 30 | 0.3 | ns | 0.11 | -0.5 | 0.93 | 21 |
| BETPE | BBCH07 | 31 | 0.5 | ns | 0.2 | -0.6 | 0.75 | 18 |
| BETPE | BBCH07 | 32 | 1.1 | ns | 0.29 | -0.35 | 0.86 | 19 |
| BETPE | BBCH07 | 34 | 1.1 | ns | 0.33 | -0.28 | 0.93 | 21 |
| BETPE | BBCH07 | 36 | 0 | ns | 0 | -0.58 | 0.64 | 19 |
| BETPE | BBCH07 | 37 | -0.2 | ns | -0.03 | -1.33 | 1.2 | 12 |
| BETPE | BBCH07 | 38 | 1.8 | ns | 0.56 | -0.1 | 1.13 | 20 |
| BETPE | BBCH07 | 39 | 1.9 | ns | 0.46 | -0.06 | 1 | 21 |
| BETPE | BBCH07 | 40 | 0 | ns | 0 | -1.5 | 1.4 | 11 |
| BETPE | BBCH07 | 41 | -0.1 | ns | 0 | -0.75 | 0.78 | 19 |

| | | | | | | | | |
|---|---|---|---|---|---|---|---|---|
| BETPE | BBCH07 | 42 | 1.7 | ns | 0.38 | -0.2 | 0.87 | 19 |
| BETPE | BBCH15 | 5 | -0.5 | ns | -0.33 | -4 | 1.5 | 10 |
| BETPE | BBCH15 | 8 | 2.1 | <0.05 | 0.82 | 0.07 | 1.5 | 19 |
| BETPE | BBCH15 | 10 | -2.2 | <0.05 | -0.84 | -1.64 | -0.15 | 20 |
| BETPE | BBCH15 | 14 | -0.9 | ns | -0.5 | -1.13 | 0.5 | 19 |
| BETPE | BBCH15 | 18 | -1.8 | ns | -0.55 | -1.25 | 0.13 | 18 |
| BETPE | BBCH15 | 19 | -0.9 | ns | -0.21 | -0.67 | 0.5 | 21 |
| BETPE | BBCH15 | 21 | -2.4 | <0.05 | -0.85 | -1.75 | -0.21 | 20 |
| BETPE | BBCH15 | 22 | -1.6 | ns | -0.5 | -1.5 | 0.29 | 15 |
| BETPE | BBCH15 | 24 | 1.5 | ns | 0.5 | -0.5 | 1.67 | 13 |
| BETPE | BBCH15 | 26 | -1.6 | ns | -1.25 | -2.6 | 0.5 | 11 |
| BETPE | BBCH15 | 28 | -1.5 | ns | -0.61 | -2 | 0.25 | 12 |
| BETPE | BBCH15 | 29 | -2.4 | <0.05 | -1.5 | -3 | -1 | 10 |
| BETPE | BBCH15 | 30 | -1.5 | ns | -0.31 | -1 | 0.23 | 20 |
| BETPE | BBCH15 | 31 | -2.1 | <0.05 | -0.75 | -1.5 | -0.14 | 18 |
| BETPE | BBCH15 | 32 | -0.2 | ns | -0.11 | -0.7 | 0.5 | 18 |
| BETPE | BBCH15 | 34 | -2.5 | <0.05 | -0.74 | -1.33 | -0.25 | 20 |
| BETPE | BBCH15 | 36 | -2.1 | <0.05 | -1 | -1.5 | -0.15 | 19 |
| BETPE | BBCH15 | 37 | 1.2 | ns | 1.25 | -1.13 | 3 | 12 |
| BETPE | BBCH15 | 38 | 3 | <0.01 | 1.33 | 0.5 | 1.9 | 20 |
| BETPE | BBCH15 | 39 | 1.7 | ns | 0.52 | -0.14 | 1.24 | 20 |
| BETPE | BBCH15 | 40 | 0.4 | ns | 0.13 | -1.5 | 2.2 | 11 |
| BETPE | BBCH15 | 41 | 1.9 | ns | 0.59 | -0.14 | 1.1 | 17 |
| BETPE | BBCH15 | 42 | 1 | ns | 0.31 | -0.33 | 0.86 | 18 |
| BETPE | BBCH61 | 19 | 0 | ns | 0 | -2 | 2.75 | 10 |
| BETPE | BBCH61 | 38 | -1.1 | ns | -0.33 | -1.29 | 1.17 | 10 |
| BETPE | BBCH61 | 39 | 0.4 | ns | 0.2 | -1.71 | 2 | 10 |
| BETPE | BBCH92 | 5 | 0 | ns | 0 | -1.57 | 0.5 | 10 |
| BETPE | BBCH92 | 8 | -2.1 | <0.05 | -0.5 | -1 | -0.07 | 18 |
| BETPE | BBCH92 | 10 | 0.1 | ns | 0 | -0.91 | 0.83 | 17 |
| BETPE | BBCH92 | 14 | -0.3 | ns | -0.07 | -0.7 | 0.67 | 18 |
| BETPE | BBCH92 | 18 | -1.6 | ns | -0.44 | -1 | 0.2 | 16 |
| BETPE | BBCH92 | 19 | -2.6 | <0.05 | -0.59 | -1.09 | -0.23 | 21 |
| BETPE | BBCH92 | 21 | -1 | ns | -0.33 | -1.13 | 0.64 | 19 |
| BETPE | BBCH92 | 22 | 1.3 | ns | 0.54 | -0.58 | 1 | 15 |
| BETPE | BBCH92 | 24 | -1.2 | ns | -0.2 | -1.33 | 0.5 | 13 |
| BETPE | BBCH92 | 30 | 0.8 | ns | 0.11 | -0.24 | 0.5 | 20 |
| BETPE | BBCH92 | 32 | -1.6 | ns | -0.46 | -1 | 0.2 | 18 |
| BETPE | BBCH92 | 34 | 1.1 | ns | 0.25 | -0.25 | 0.67 | 21 |
| BETPE | BBCH92 | 36 | -1.8 | ns | -0.56 | -1.08 | 0.2 | 16 |
| BETPE | BBCH92 | 37 | -1 | ns | -0.67 | -2.18 | 1 | 12 |
| BETPE | BBCH92 | 38 | 1.4 | ns | 0.4 | -0.25 | 1.07 | 20 |
| BETPE | BBCH92 | 39 | -2 | ns | -0.31 | -0.79 | -0.07 | 21 |
| BETPE | BBCH92 | 41 | -1.3 | ns | -0.38 | -1.17 | 0.29 | 17 |
| BETPE | BBCH92 | 42 | 0.3 | ns | 0.14 | -0.8 | 0.93 | 17 |
| BETPE | BBCH97 | 5 | 0.1 | ns | 0 | -1.8 | 2 | 10 |
| BETPE | BBCH97 | 8 | -1.3 | ns | -0.35 | -0.91 | 0.25 | 17 |
| BETPE | BBCH97 | 10 | -0.4 | ns | -0.09 | -0.86 | 0.75 | 16 |

| | | | | | | | | |
|---|---|---|---|---|---|---|---|---|
| BETPE | BBCH97 | 14 | -0.3 | ns | 0 | -0.67 | 0.33 | 18 |
| BETPE | BBCH97 | 18 | 0.3 | ns | 0.08 | -0.55 | 0.5 | 14 |
| BETPE | BBCH97 | 19 | -0.3 | ns | -0.03 | -0.5 | 0.39 | 20 |
| BETPE | BBCH97 | 21 | -0.8 | ns | -0.28 | -1.87 | 1 | 17 |
| BETPE | BBCH97 | 22 | 0.3 | ns | 0.23 | -0.86 | 1.33 | 13 |
| BETPE | BBCH97 | 24 | -0.7 | ns | -0.25 | -1.6 | 0.5 | 13 |
| BETPE | BBCH97 | 30 | 1.2 | ns | 0.23 | -0.25 | 0.6 | 19 |
| BETPE | BBCH97 | 32 | -2.5 | <0.05 | -0.58 | -1.56 | -0.21 | 18 |
| BETPE | BBCH97 | 34 | 0.7 | ns | 0.18 | -0.38 | 0.64 | 20 |
| BETPE | BBCH97 | 36 | -1.6 | ns | -0.5 | -1 | 0.27 | 15 |
| BETPE | BBCH97 | 37 | 1.2 | ns | 0.56 | -1.25 | 3 | 11 |
| BETPE | BBCH97 | 38 | 0.7 | ns | 0.33 | -0.67 | 1 | 19 |
| BETPE | BBCH97 | 39 | -2.1 | <0.05 | -0.55 | -1.09 | -0.07 | 21 |
| BETPE | BBCH97 | 41 | -1.3 | ns | -0.44 | -1.19 | 0.4 | 17 |
| BETPE | BBCH97 | 42 | -1.7 | ns | -0.58 | -1.63 | 0.17 | 12 |
| IUPCO | BBCH61 | 1 | -0.3 | ns | -0.13 | -1.17 | 0.91 | 20 |
| IUPCO | BBCH61 | 6 | -0.6 | ns | -0.56 | -1.4 | 1.17 | 10 |
| IUPCO | BBCH61 | 10 | -0.6 | ns | -0.26 | -1.25 | 0.44 | 17 |
| IUPCO | BBCH61 | 14 | 0.4 | ns | 0.11 | -0.6 | 1 | 17 |
| IUPCO | BBCH61 | 18 | -2.5 | <0.05 | -1 | -1.89 | -0.2 | 14 |
| IUPCO | BBCH61 | 19 | -2.6 | <0.05 | -0.88 | -1.26 | -0.33 | 20 |
| IUPCO | BBCH61 | 21 | -1.4 | ns | -0.4 | -1 | 0.29 | 18 |
| IUPCO | BBCH61 | 22 | 0 | ns | 0.14 | -2 | 1.75 | 10 |
| IUPCO | BBCH61 | 24 | 1.1 | ns | 0.35 | -1 | 2 | 12 |
| IUPCO | BBCH61 | 30 | -3.2 | <0.01 | -0.74 | -1.13 | -0.4 | 20 |
| IUPCO | BBCH61 | 31 | -1.6 | ns | -0.59 | -1.22 | 0.2 | 12 |
| IUPCO | BBCH61 | 32 | 0 | ns | 0 | -0.8 | 0.55 | 16 |
| IUPCO | BBCH61 | 34 | -2.9 | <0.01 | -0.8 | -1.25 | -0.33 | 20 |
| IUPCO | BBCH61 | 36 | -2.2 | <0.05 | -0.7 | -1.44 | -0.2 | 15 |
| IUPCO | BBCH61 | 37 | -0.3 | ns | -0.5 | -2 | 1.17 | 10 |
| IUPCO | BBCH61 | 38 | -1.6 | ns | -0.57 | -0.94 | 0.2 | 18 |
| IUPCO | BBCH61 | 39 | -1.5 | ns | -0.33 | -0.85 | 0.2 | 20 |
| IUPCO | BBCH61 | 40 | 0 | ns | 0 | -1 | 1 | 10 |
| IUPCO | BBCH61 | 41 | 0 | ns | 0 | -0.78 | 0.8 | 17 |
| IUPCO | BBCH61 | 42 | -2.3 | <0.05 | -0.44 | -1 | -0.1 | 16 |
| PIEAB | BBCH30 | 1 | -1.8 | ns | -0.86 | -3 | 0.5 | 11 |
| PIEAB | BBCH30 | 5 | -1.4 | ns | -0.6 | -2 | 0.5 | 10 |
| PIEAB | BBCH30 | 6 | -1.8 | ns | -1 | -2.4 | 0.2 | 14 |
| PIEAB | BBCH30 | 8 | -1.2 | ns | -0.27 | -0.92 | 0.33 | 19 |
| PIEAB | BBCH30 | 10 | -3.6 | <0.001 | -0.92 | -1.33 | -0.5 | 20 |
| PIEAB | BBCH30 | 14 | -1.6 | ns | -0.5 | -1 | 0.32 | 19 |
| PIEAB | BBCH30 | 18 | -3.5 | <0.01 | -0.93 | -1.33 | -0.5 | 17 |
| PIEAB | BBCH30 | 19 | -1.2 | ns | -0.5 | -1.4 | 0.33 | 21 |
| PIEAB | BBCH30 | 21 | 0.1 | ns | 0 | -0.75 | 0.67 | 21 |
| PIEAB | BBCH30 | 22 | -0.4 | ns | -0.3 | -1.5 | 0.57 | 15 |
| PIEAB | BBCH30 | 24 | -0.1 | ns | -0.06 | -1.86 | 1.67 | 13 |
| PIEAB | BBCH30 | 26 | -1.7 | ns | -1.86 | -4.5 | 0.5 | 11 |
| PIEAB | BBCH30 | 28 | -1.1 | ns | -0.75 | -2.8 | 0.56 | 14 |

| | | | | | | | | |
|---|---|---|---|---|---|---|---|---|
| PIEAB | BBCH30 | 29 | -2.7 | <0.05 | -3 | -4.4 | -2 | 10 |
| PIEAB | BBCH30 | 30 | -2 | ns | -0.5 | -1.2 | -0.07 | 20 |
| PIEAB | BBCH30 | 31 | -2.3 | <0.05 | -1.57 | -2.63 | -0.5 | 17 |
| PIEAB | BBCH30 | 32 | -0.9 | ns | -0.39 | -1.25 | 0.33 | 18 |
| PIEAB | BBCH30 | 34 | -3.1 | <0.01 | -0.88 | -1.5 | -0.54 | 20 |
| PIEAB | BBCH30 | 36 | -2.1 | <0.05 | -0.83 | -1.2 | -0.18 | 18 |
| PIEAB | BBCH30 | 37 | -2.3 | <0.05 | -1.78 | -4 | -0.25 | 12 |
| PIEAB | BBCH30 | 38 | -2.1 | <0.05 | -0.67 | -1.33 | -0.07 | 20 |
| PIEAB | BBCH30 | 39 | -0.2 | ns | 0 | -0.7 | 0.6 | 20 |
| PIEAB | BBCH30 | 40 | -0.9 | ns | -0.6 | -2.33 | 1 | 11 |
| PIEAB | BBCH30 | 41 | -1.7 | ns | -0.67 | -1.67 | 0.33 | 19 |
| PIEAB | BBCH30 | 42 | -2.1 | <0.05 | -0.63 | -1.5 | -0.09 | 19 |
| PIEAB | BBCH39 | 1 | -2.2 | <0.05 | -1.67 | -3.5 | -0.33 | 11 |
| PIEAB | BBCH39 | 5 | -1.4 | ns | -1 | -2 | 0.33 | 10 |
| PIEAB | BBCH39 | 6 | 0.3 | ns | 0.18 | -0.71 | 1.33 | 14 |
| PIEAB | BBCH39 | 8 | -0.2 | ns | -0.11 | -1 | 0.69 | 18 |
| PIEAB | BBCH39 | 10 | -2 | ns | -1.44 | -2.8 | -0.25 | 11 |
| PIEAB | BBCH39 | 14 | 0.5 | ns | 0.17 | -0.67 | 1.2 | 18 |
| PIEAB | BBCH39 | 18 | -1.2 | ns | -0.47 | -1.3 | 0.5 | 17 |
| PIEAB | BBCH39 | 19 | 1.1 | ns | 0.5 | -0.41 | 1.33 | 20 |
| PIEAB | BBCH39 | 21 | -1.2 | ns | -0.33 | -0.83 | 0.33 | 20 |
| PIEAB | BBCH39 | 22 | 2.3 | <0.05 | 1.33 | 0.33 | 2 | 15 |
| PIEAB | BBCH39 | 24 | 1 | ns | 0.5 | -0.6 | 1.9 | 13 |
| PIEAB | BBCH39 | 26 | -2.7 | <0.05 | -3.75 | -6 | -1.25 | 10 |
| PIEAB | BBCH39 | 28 | -0.6 | ns | -0.5 | -2.33 | 1.38 | 10 |
| PIEAB | BBCH39 | 30 | -0.7 | ns | -0.21 | -1 | 0.46 | 19 |
| PIEAB | BBCH39 | 31 | 1.1 | ns | 0.4 | -0.56 | 1.5 | 14 |
| PIEAB | BBCH39 | 32 | -2.1 | <0.05 | -0.57 | -1.2 | -0.08 | 16 |
| PIEAB | BBCH39 | 34 | -2.5 | <0.05 | -1.24 | -1.92 | -0.38 | 20 |
| PIEAB | BBCH39 | 36 | -0.1 | ns | -0.19 | -1 | 1 | 15 |
| PIEAB | BBCH39 | 37 | 0.7 | ns | 1.13 | -1 | 3.5 | 11 |
| PIEAB | BBCH39 | 38 | 2.4 | <0.05 | 0.54 | 0.29 | 1 | 20 |
| PIEAB | BBCH39 | 39 | -1 | ns | -0.19 | -0.67 | 0.27 | 20 |
| PIEAB | BBCH39 | 41 | 2.5 | <0.05 | 0.92 | 0.42 | 1.67 | 16 |
| PIEAB | BBCH39 | 42 | 0.8 | ns | 0.33 | -2 | 1.39 | 10 |
| PIEAB | BBCH61 | 5 | -0.9 | ns | -0.83 | -3.4 | 1.17 | 10 |
| PIEAB | BBCH61 | 5 | -1.6 | ns | -1.22 | -3.5 | 1 | 10 |
| PIEAB | BBCH61 | 6 | -1.8 | ns | -0.59 | -1.33 | 0.2 | 13 |
| PIEAB | BBCH61 | 6 | 0 | ns | 0 | -1.5 | 1 | 13 |
| PIEAB | BBCH61 | 8 | -1.8 | ns | -1.24 | -2.11 | 0.15 | 16 |
| PIEAB | BBCH61 | 8 | -2.3 | <0.05 | -1.21 | -2.83 | -0.22 | 12 |
| PIEAB | BBCH61 | 10 | -2.5 | <0.05 | -0.8 | -1.88 | -0.17 | 17 |
| PIEAB | BBCH61 | 10 | -1.5 | ns | -0.5 | -1.26 | 0.5 | 13 |
| PIEAB | BBCH61 | 14 | -0.7 | ns | -0.5 | -1.58 | 1 | 14 |
| PIEAB | BBCH61 | 14 | -0.4 | ns | -0.33 | -1.27 | 1 | 14 |
| PIEAB | BBCH61 | 18 | -0.7 | ns | -0.33 | -1.57 | 0.75 | 13 |
| PIEAB | BBCH61 | 18 | -0.7 | ns | -0.15 | -1.17 | 1 | 13 |
| PIEAB | BBCH61 | 19 | 0.3 | ns | 0.27 | -1.22 | 1.33 | 13 |

| | | | | | | | | |
|---|---|---|---|---|---|---|---|---|
| PIEAB | BBCH61 | 19 | -0.3 | ns | -0.24 | -1.44 | 1.23 | 10 |
| PIEAB | BBCH61 | 21 | -0.7 | ns | -0.17 | -0.83 | 0.67 | 14 |
| PIEAB | BBCH61 | 24 | 1.2 | ns | 1.5 | -1.33 | 4 | 10 |
| PIEAB | BBCH61 | 28 | -1.9 | ns | -0.5 | -1.91 | 0.29 | 11 |
| PIEAB | BBCH61 | 28 | -0.3 | ns | -0.2 | -1 | 0.71 | 10 |
| PIEAB | BBCH61 | 30 | -0.5 | ns | -0.13 | -1 | 0.71 | 18 |
| PIEAB | BBCH61 | 30 | -0.2 | ns | 0 | -1 | 1.5 | 15 |
| PIEAB | BBCH61 | 31 | 0 | ns | 0 | -0.75 | 1.17 | 13 |
| PIEAB | BBCH61 | 31 | 0.6 | ns | 0.13 | -1 | 1.33 | 10 |
| PIEAB | BBCH61 | 32 | 1.5 | ns | 0.71 | -0.25 | 1.5 | 13 |
| PIEAB | BBCH61 | 32 | 0.7 | ns | 0.33 | -0.5 | 1 | 10 |
| PIEAB | BBCH61 | 34 | 0.9 | ns | 0.21 | -0.53 | 1 | 17 |
| PIEAB | BBCH61 | 34 | -0.4 | ns | -0.07 | -1.17 | 0.8 | 14 |
| PIEAB | BBCH61 | 38 | -1.3 | ns | -0.47 | -1.5 | 0.33 | 13 |
| PIEAB | BBCH61 | 38 | -0.5 | ns | -0.34 | -1.5 | 0.67 | 12 |
| PIEAB | BBCH61 | 39 | 1 | ns | 0.31 | -1 | 1.4 | 13 |
| PIEAB | BBCH61 | 39 | 0.5 | ns | 0.1 | -0.67 | 0.64 | 12 |
| PIEAB | BBCH61 | 41 | -0.2 | ns | -0.1 | -1.25 | 0.8 | 13 |
| PIEAB | BBCH61 | 41 | 1.6 | ns | 0.67 | -0.5 | 1.67 | 14 |
| PIEAB | BBCH61 | 42 | 1.5 | ns | 0.86 | -0.5 | 2.25 | 11 |
| PIEAB | BBCH63 | 5 | -1.3 | ns | -1 | -3 | 0.67 | 10 |
| PIEAB | BBCH63 | 6 | -2 | ns | -1.43 | -3.6 | -0.5 | 11 |
| PIEAB | BBCH63 | 8 | -1.7 | ns | -1.28 | -2.8 | 0.44 | 13 |
| PIEAB | BBCH63 | 10 | -1.7 | ns | -0.44 | -1 | 0.27 | 15 |
| PIEAB | BBCH63 | 14 | 0.3 | ns | 0.15 | -1.25 | 1.71 | 13 |
| PIEAB | BBCH63 | 18 | -0.1 | ns | -0.04 | -1 | 1 | 13 |
| PIEAB | BBCH63 | 19 | -1.3 | ns | -0.7 | -2.67 | 0.42 | 12 |
| PIEAB | BBCH63 | 21 | -1.7 | ns | -0.67 | -1.5 | 0.3 | 12 |
| PIEAB | BBCH63 | 28 | -2.1 | <0.05 | -1 | -2.75 | -0.25 | 10 |
| PIEAB | BBCH63 | 30 | -0.4 | ns | -0.24 | -1 | 0.71 | 16 |
| PIEAB | BBCH63 | 31 | -0.9 | ns | -0.2 | -1.13 | 0.67 | 11 |
| PIEAB | BBCH63 | 32 | 1 | ns | 0.5 | -0.31 | 1.33 | 11 |
| PIEAB | BBCH63 | 34 | -1.3 | ns | -0.59 | -1.3 | 0.33 | 17 |
| PIEAB | BBCH63 | 38 | -0.3 | ns | -0.1 | -1.25 | 0.83 | 12 |
| PIEAB | BBCH63 | 39 | 1.2 | ns | 0.42 | -0.33 | 1 | 13 |
| PIEAB | BBCH63 | 41 | 0.4 | ns | 0.16 | -1 | 0.8 | 13 |
| PIUSI | BBCH30 | 1 | -1.6 | ns | -0.5 | -1.29 | 0.17 | 18 |
| PIUSI | BBCH30 | 3 | -2.8 | <0.01 | -1 | -1.53 | -0.5 | 20 |
| PIUSI | BBCH30 | 5 | -2.7 | <0.05 | -3 | -4 | -1.29 | 10 |
| PIUSI | BBCH30 | 6 | -3 | <0.01 | -1.78 | -2.86 | -0.71 | 14 |
| PIUSI | BBCH30 | 8 | -1.2 | ns | -0.52 | -1.29 | 0.33 | 20 |
| PIUSI | BBCH30 | 10 | -2.6 | <0.05 | -1.2 | -1.81 | -0.63 | 20 |
| PIUSI | BBCH30 | 14 | 0.1 | ns | 0 | -1 | 1 | 19 |
| PIUSI | BBCH30 | 18 | -0.4 | ns | -0.08 | -1.07 | 0.79 | 18 |
| PIUSI | BBCH30 | 19 | -1.1 | ns | -0.35 | -1.13 | 0.5 | 21 |
| PIUSI | BBCH30 | 21 | -0.4 | ns | -0.11 | -0.71 | 0.5 | 21 |
| PIUSI | BBCH30 | 22 | -0.9 | ns | -0.5 | -1.75 | 1.2 | 15 |
| PIUSI | BBCH30 | 24 | -1.2 | ns | -0.84 | -2.78 | 0.71 | 13 |

| | | | | | | | | |
|---|---|---|---|---|---|---|---|---|
| PIUSI | BBCH30 | 26 | -0.5 | ns | -0.75 | -3.2 | 2.5 | 11 |
| PIUSI | BBCH30 | 28 | -0.4 | ns | -0.2 | -1.67 | 1.5 | 14 |
| PIUSI | BBCH30 | 29 | -2.1 | <0.05 | -1.25 | -2.6 | -0.14 | 10 |
| PIUSI | BBCH30 | 30 | -1.1 | ns | -0.25 | -0.8 | 0.2 | 20 |
| PIUSI | BBCH30 | 31 | -2 | ns | -0.33 | -1 | -0.08 | 18 |
| PIUSI | BBCH30 | 32 | -1.9 | ns | -0.4 | -1 | 0.08 | 18 |
| PIUSI | BBCH30 | 34 | 0 | ns | 0 | -0.83 | 0.8 | 20 |
| PIUSI | BBCH30 | 36 | 3.4 | <0.01 | 1.3 | 0.78 | 1.81 | 18 |
| PIUSI | BBCH30 | 37 | -1.8 | ns | -1.06 | -2.33 | 0.2 | 13 |
| PIUSI | BBCH30 | 38 | -1.4 | ns | -0.38 | -1.2 | 0.29 | 20 |
| PIUSI | BBCH30 | 39 | 0 | ns | 0 | -0.5 | 0.5 | 20 |
| PIUSI | BBCH30 | 40 | -1.4 | ns | -0.67 | -1.83 | 0.29 | 11 |
| PIUSI | BBCH30 | 41 | -0.3 | ns | -0.11 | -1 | 0.6 | 19 |
| PIUSI | BBCH30 | 42 | -1.5 | ns | -0.5 | -1.25 | 0.25 | 18 |
| PIUSI | BBCH39 | 1 | -1.1 | ns | -0.26 | -1.06 | 0.33 | 20 |
| PIUSI | BBCH39 | 3 | -1.8 | ns | -1.06 | -2.5 | 0.14 | 18 |
| PIUSI | BBCH39 | 5 | -0.9 | ns | -0.6 | -2.2 | 1.33 | 10 |
| PIUSI | BBCH39 | 6 | -0.7 | ns | -0.3 | -1.33 | 0.67 | 14 |
| PIUSI | BBCH39 | 8 | 1.8 | ns | 0.8 | -0.17 | 1.57 | 18 |
| PIUSI | BBCH39 | 10 | -0.9 | ns | -0.17 | -1 | 0.5 | 15 |
| PIUSI | BBCH39 | 14 | 1.3 | ns | 0.43 | -0.43 | 1.8 | 19 |
| PIUSI | BBCH39 | 18 | 0.6 | ns | 0.24 | -0.67 | 1.29 | 17 |
| PIUSI | BBCH39 | 19 | 1.2 | ns | 0.44 | -0.39 | 1.33 | 20 |
| PIUSI | BBCH39 | 21 | -0.5 | ns | -0.06 | -0.5 | 0.36 | 21 |
| PIUSI | BBCH39 | 22 | 1.9 | ns | 0.86 | -0.2 | 1.78 | 15 |
| PIUSI | BBCH39 | 24 | -0.9 | ns | -0.17 | -0.78 | 0.5 | 13 |
| PIUSI | BBCH39 | 26 | -1.2 | ns | -1.75 | -5 | 2 | 10 |
| PIUSI | BBCH39 | 30 | -1.1 | ns | -0.34 | -1 | 0.2 | 20 |
| PIUSI | BBCH39 | 31 | -0.4 | ns | -0.2 | -1.5 | 1.38 | 14 |
| PIUSI | BBCH39 | 32 | 2.1 | <0.05 | 0.5 | 0.11 | 1.13 | 17 |
| PIUSI | BBCH39 | 34 | -1.8 | ns | -1.2 | -2.42 | 0.18 | 20 |
| PIUSI | BBCH39 | 36 | 1 | ns | 0.73 | -1 | 1.67 | 17 |
| PIUSI | BBCH39 | 37 | 1.1 | ns | 1.2 | -2 | 3.67 | 11 |
| PIUSI | BBCH39 | 38 | 0.2 | ns | 0.09 | -0.67 | 0.88 | 20 |
| PIUSI | BBCH39 | 39 | 1.5 | ns | 0.43 | -0.25 | 1 | 20 |
| PIUSI | BBCH39 | 40 | 1.7 | ns | 1.25 | -0.75 | 2.5 | 10 |
| PIUSI | BBCH39 | 41 | 0.3 | ns | 0.09 | -0.56 | 0.89 | 16 |
| PIUSI | BBCH39 | 42 | 1.3 | ns | 0.77 | -0.67 | 2 | 14 |
| PIUSI | BBCH61 | 1 | -0.9 | ns | -0.31 | -1.29 | 0.46 | 19 |
| PIUSI | BBCH61 | 1 | 0.7 | ns | 0.26 | -0.5 | 1 | 20 |
| PIUSI | BBCH61 | 3 | -1.9 | ns | -0.6 | -1.67 | 0.2 | 18 |
| PIUSI | BBCH61 | 3 | -0.9 | ns | -0.4 | -1.43 | 0.57 | 19 |
| PIUSI | BBCH61 | 5 | -1.3 | ns | -0.67 | -2.33 | 1 | 10 |
| PIUSI | BBCH61 | 5 | -1.9 | ns | -0.83 | -2.67 | 0.5 | 10 |
| PIUSI | BBCH61 | 6 | -1.8 | ns | -0.55 | -1.2 | 0.17 | 13 |
| PIUSI | BBCH61 | 6 | -1 | ns | -0.19 | -1.25 | 0.5 | 13 |
| PIUSI | BBCH61 | 8 | -0.5 | ns | -0.29 | -1.78 | 0.88 | 15 |
| PIUSI | BBCH61 | 8 | -0.6 | ns | -0.28 | -1.5 | 0.88 | 16 |

| | | | | | | | | |
|---|---|---|---|---|---|---|---|---|
| PIUSI | BBCH61 | 10 | -2.1 | <0.05 | -0.56 | -1.17 | -0.13 | 19 |
| PIUSI | BBCH61 | 10 | -2.1 | <0.05 | -0.75 | -1.39 | -0.13 | 19 |
| PIUSI | BBCH61 | 14 | -1.4 | ns | -0.75 | -1.33 | 0.33 | 18 |
| PIUSI | BBCH61 | 14 | -0.8 | ns | -0.5 | -1.25 | 0.75 | 17 |
| PIUSI | BBCH61 | 18 | -2.5 | <0.05 | -0.93 | -1.9 | -0.4 | 17 |
| PIUSI | BBCH61 | 18 | -2.6 | <0.05 | -1 | -1.71 | -0.33 | 18 |
| PIUSI | BBCH61 | 19 | -1.6 | ns | -0.37 | -1 | 0.17 | 21 |
| PIUSI | BBCH61 | 19 | -1.5 | ns | -0.29 | -1 | 0.25 | 21 |
| PIUSI | BBCH61 | 21 | -3.1 | <0.01 | -1 | -1.55 | -0.5 | 20 |
| PIUSI | BBCH61 | 21 | -2.6 | <0.05 | -0.71 | -1.29 | -0.25 | 21 |
| PIUSI | BBCH61 | 22 | -1.5 | ns | -0.64 | -1.25 | 0.33 | 14 |
| PIUSI | BBCH61 | 22 | -2.6 | <0.05 | -0.8 | -1.33 | -0.43 | 14 |
| PIUSI | BBCH61 | 24 | -0.5 | ns | -0.28 | -1.22 | 0.86 | 13 |
| PIUSI | BBCH61 | 24 | -0.2 | ns | 0 | -1 | 0.5 | 13 |
| PIUSI | BBCH61 | 26 | -0.9 | ns | -0.5 | -1.29 | 1 | 11 |
| PIUSI | BBCH61 | 26 | -1.3 | ns | -0.5 | -2 | 0.5 | 11 |
| PIUSI | BBCH61 | 28 | -1.6 | ns | -0.91 | -2.83 | 0.5 | 11 |
| PIUSI | BBCH61 | 28 | -0.8 | ns | -0.44 | -2.4 | 0.67 | 12 |
| PIUSI | BBCH61 | 29 | 0.2 | ns | 0.2 | -2 | 2.5 | 10 |
| PIUSI | BBCH61 | 29 | -0.1 | ns | -0.2 | -2.33 | 2.2 | 10 |
| PIUSI | BBCH61 | 30 | -2.6 | <0.05 | -0.65 | -1.11 | -0.25 | 20 |
| PIUSI | BBCH61 | 30 | -2.6 | <0.05 | -0.67 | -1.09 | -0.25 | 20 |
| PIUSI | BBCH61 | 31 | -1.5 | ns | -0.43 | -1 | 0.22 | 18 |
| PIUSI | BBCH61 | 31 | -2.7 | <0.05 | -0.86 | -1.33 | -0.38 | 17 |
| PIUSI | BBCH61 | 32 | -2.6 | <0.05 | -0.59 | -1.17 | -0.2 | 19 |
| PIUSI | BBCH61 | 32 | -2.2 | <0.05 | -0.67 | -1.14 | -0.13 | 18 |
| PIUSI | BBCH61 | 34 | -2 | ns | -0.46 | -1 | -0.09 | 19 |
| PIUSI | BBCH61 | 34 | -2.6 | <0.05 | -0.5 | -1 | -0.17 | 19 |
| PIUSI | BBCH61 | 36 | -1.9 | ns | -0.89 | -1.78 | 0.17 | 12 |
| PIUSI | BBCH61 | 36 | -1.8 | ns | -0.78 | -1.67 | 0.25 | 13 |
| PIUSI | BBCH61 | 37 | 0 | ns | 0 | -1.83 | 2 | 10 |
| PIUSI | BBCH61 | 37 | -0.5 | ns | -0.4 | -2.5 | 1.8 | 10 |
| PIUSI | BBCH61 | 38 | -2.2 | <0.05 | -0.55 | -1 | -0.17 | 20 |
| PIUSI | BBCH61 | 38 | -2.5 | <0.05 | -0.78 | -1.22 | -0.4 | 19 |
| PIUSI | BBCH61 | 39 | -0.6 | ns | -0.12 | -0.83 | 0.56 | 20 |
| PIUSI | BBCH61 | 39 | -0.4 | ns | -0.07 | -0.75 | 0.5 | 20 |
| PIUSI | BBCH61 | 40 | -0.9 | ns | -0.57 | -2.2 | 0.67 | 11 |
| PIUSI | BBCH61 | 40 | -0.8 | ns | -0.4 | -2 | 1 | 10 |
| PIUSI | BBCH61 | 41 | -1.3 | ns | -0.33 | -0.89 | 0.36 | 18 |
| PIUSI | BBCH61 | 41 | -2 | ns | -0.62 | -1 | -0.13 | 19 |
| PIUSI | BBCH61 | 42 | -0.4 | ns | -0.04 | -0.75 | 0.5 | 16 |
| PIUSI | BBCH61 | 42 | -0.9 | ns | -0.2 | -0.6 | 0.3 | 14 |
| PIUSI | BBCH63 | 1 | -0.6 | ns | -0.23 | -1.17 | 0.83 | 17 |
| PIUSI | BBCH63 | 3 | -3.8 | <0.001 | -1.82 | -2.56 | -1.25 | 15 |
| PIUSI | BBCH63 | 5 | -1.3 | ns | -0.86 | -3 | 0.6 | 10 |
| PIUSI | BBCH63 | 6 | -1.9 | ns | -1.43 | -2.17 | 0.13 | 11 |
| PIUSI | BBCH63 | 8 | 0 | ns | 0 | -1.54 | 1.62 | 13 |
| PIUSI | BBCH63 | 10 | -1.2 | ns | -0.46 | -1.29 | 0.5 | 16 |

| | | | | | | | | |
|---|---|---|---|---|---|---|---|---|
| PIUSI | BBCH63 | 14 | -0.9 | ns | -0.45 | -1 | 0.71 | 16 |
| PIUSI | BBCH63 | 18 | -1.9 | ns | -0.79 | -1.63 | 0.09 | 18 |
| PIUSI | BBCH63 | 19 | -2.6 | <0.05 | -0.79 | -1.25 | -0.29 | 20 |
| PIUSI | BBCH63 | 21 | -2.4 | <0.05 | -0.68 | -1.54 | -0.2 | 19 |
| PIUSI | BBCH63 | 22 | -1.5 | ns | -0.8 | -2.13 | 0.33 | 14 |
| PIUSI | BBCH63 | 24 | -0.8 | ns | -0.27 | -1.25 | 0.75 | 13 |
| PIUSI | BBCH63 | 26 | -0.4 | ns | -0.4 | -2 | 1 | 11 |
| PIUSI | BBCH63 | 28 | -1.5 | ns | -0.88 | -2 | 0.44 | 10 |
| PIUSI | BBCH63 | 30 | -3 | <0.01 | -0.79 | -1.25 | -0.29 | 20 |
| PIUSI | BBCH63 | 31 | -2.2 | <0.05 | -0.5 | -1.38 | -0.17 | 14 |
| PIUSI | BBCH63 | 32 | -2 | ns | -0.57 | -1.33 | -0.1 | 18 |
| PIUSI | BBCH63 | 34 | -2.8 | <0.01 | -0.67 | -1.14 | -0.36 | 19 |
| PIUSI | BBCH63 | 36 | -1.3 | ns | -1.17 | -2 | 1 | 11 |
| PIUSI | BBCH63 | 37 | 1.1 | ns | 0.5 | -1 | 1.71 | 10 |
| PIUSI | BBCH63 | 38 | -1.3 | ns | -0.25 | -0.65 | 0.25 | 19 |
| PIUSI | BBCH63 | 39 | -1.3 | ns | -0.35 | -0.75 | 0.25 | 20 |
| PIUSI | BBCH63 | 40 | -0.6 | ns | -0.67 | -3 | 1.5 | 10 |
| PIUSI | BBCH63 | 41 | -1.1 | ns | -0.31 | -0.8 | 0.3 | 19 |
| PIUSI | BBCH63 | 42 | -0.8 | ns | -0.17 | -0.83 | 0.38 | 16 |
| POPTR | BBCH15 | 1 | -2.2 | <0.05 | -0.68 | -1.33 | -0.13 | 21 |
| POPTR | BBCH15 | 2 | -0.7 | ns | -0.63 | -2.67 | 2 | 11 |
| POPTR | BBCH15 | 3 | -0.9 | ns | -0.25 | -1.13 | 0.33 | 18 |
| POPTR | BBCH15 | 5 | -1.1 | ns | -0.83 | -3.5 | 1 | 10 |
| POPTR | BBCH15 | 8 | 1.3 | ns | 0.75 | -0.29 | 1.67 | 17 |
| POPTR | BBCH15 | 10 | -1.7 | ns | -0.57 | -1.25 | 0.17 | 20 |
| POPTR | BBCH15 | 14 | -0.2 | ns | -0.12 | -0.91 | 0.67 | 19 |
| POPTR | BBCH15 | 18 | -1.6 | ns | -0.55 | -1.2 | 0.2 | 18 |
| POPTR | BBCH15 | 19 | -1.5 | ns | -0.37 | -0.92 | 0.29 | 20 |
| POPTR | BBCH15 | 21 | -1.9 | ns | -0.62 | -1.27 | 0.08 | 21 |
| POPTR | BBCH15 | 22 | 0 | ns | 0 | -1 | 0.71 | 15 |
| POPTR | BBCH15 | 24 | 1.9 | ns | 0.5 | -0.17 | 1.11 | 13 |
| POPTR | BBCH15 | 26 | -1.1 | ns | -0.8 | -2.5 | 1.6 | 10 |
| POPTR | BBCH15 | 28 | -0.9 | ns | -1.17 | -2.71 | 0.78 | 10 |
| POPTR | BBCH15 | 29 | -1.3 | ns | -1.2 | -2.75 | 0.67 | 10 |
| POPTR | BBCH15 | 30 | -2.4 | <0.05 | -0.5 | -1 | -0.17 | 20 |
| POPTR | BBCH15 | 31 | -2.2 | <0.05 | -1 | -2 | -0.2 | 15 |
| POPTR | BBCH15 | 32 | -1.2 | ns | -0.33 | -1 | 0.38 | 19 |
| POPTR | BBCH15 | 34 | -0.8 | ns | -0.24 | -1 | 0.43 | 20 |
| POPTR | BBCH15 | 36 | -2.5 | <0.05 | -0.71 | -1.25 | -0.2 | 19 |
| POPTR | BBCH15 | 37 | 0.9 | ns | 0.5 | -1 | 1.67 | 11 |
| POPTR | BBCH15 | 38 | 2.1 | <0.05 | 0.8 | 0.25 | 1.77 | 19 |
| POPTR | BBCH15 | 39 | 0.4 | ns | 0.08 | -0.67 | 0.75 | 20 |
| POPTR | BBCH15 | 40 | 1 | ns | 0.5 | -1 | 2.25 | 11 |
| POPTR | BBCH15 | 41 | 0.5 | ns | 0.23 | -0.42 | 1 | 16 |
| POPTR | BBCH15 | 42 | -0.4 | ns | -0.08 | -1 | 0.56 | 18 |
| POPTR | BBCH92 | 1 | 1.8 | ns | 0.41 | -0.09 | 1.11 | 20 |
| POPTR | BBCH92 | 2 | 0.6 | ns | 1 | -2.67 | 2.83 | 11 |
| POPTR | BBCH92 | 3 | -0.4 | ns | -0.09 | -0.6 | 0.5 | 19 |

| | | | | | | | | |
|---|---|---|---|---|---|---|---|---|
| POPTR | BBCH92 | 5 | 1.7 | ns | 1 | -0.75 | 2.5 | 10 |
| POPTR | BBCH92 | 8 | -0.6 | ns | -0.27 | -1 | 0.53 | 17 |
| POPTR | BBCH92 | 10 | 0 | ns | 0 | -0.79 | 0.8 | 17 |
| POPTR | BBCH92 | 14 | 0.9 | ns | 0.29 | -0.4 | 1 | 18 |
| POPTR | BBCH92 | 18 | 0.6 | ns | 0.17 | -0.6 | 1 | 17 |
| POPTR | BBCH92 | 19 | 2.3 | <0.05 | 0.91 | 0.19 | 1.58 | 21 |
| POPTR | BBCH92 | 21 | -0.6 | ns | -0.14 | -1.15 | 0.75 | 19 |
| POPTR | BBCH92 | 22 | 2.1 | <0.05 | 0.78 | 0.14 | 2 | 13 |
| POPTR | BBCH92 | 24 | -1 | ns | -0.47 | -1.4 | 0.5 | 13 |
| POPTR | BBCH92 | 30 | 0 | ns | 0 | -0.75 | 0.71 | 16 |
| POPTR | BBCH92 | 32 | -1 | ns | -0.39 | -1 | 0.33 | 18 |
| POPTR | BBCH92 | 34 | 0.6 | ns | 0.09 | -0.36 | 0.5 | 19 |
| POPTR | BBCH92 | 36 | -2.6 | <0.05 | -0.71 | -1 | -0.4 | 15 |
| POPTR | BBCH92 | 37 | -0.5 | ns | -0.33 | -1.5 | 1.33 | 12 |
| POPTR | BBCH92 | 38 | 2.1 | <0.05 | 0.46 | 0.15 | 1 | 20 |
| POPTR | BBCH92 | 39 | -1.8 | ns | -0.38 | -1 | 0.09 | 20 |
| POPTR | BBCH92 | 41 | -0.1 | ns | 0 | -0.85 | 0.86 | 16 |
| POPTR | BBCH92 | 42 | -1.7 | ns | -0.4 | -1.07 | 0.14 | 15 |
| POPTR | BBCH97 | 1 | 2.3 | <0.05 | 0.8 | 0.25 | 1.33 | 19 |
| POPTR | BBCH97 | 3 | -0.2 | ns | -0.2 | -1.36 | 1 | 15 |
| POPTR | BBCH97 | 5 | 1 | ns | 0.71 | -1.4 | 3 | 10 |
| POPTR | BBCH97 | 8 | -0.8 | ns | -0.16 | -0.82 | 0.4 | 16 |
| POPTR | BBCH97 | 10 | -1.4 | ns | -0.33 | -0.82 | 0.17 | 15 |
| POPTR | BBCH97 | 14 | 1.5 | ns | 0.4 | -0.25 | 0.88 | 18 |
| POPTR | BBCH97 | 18 | 1.2 | ns | 0.4 | -0.5 | 1 | 15 |
| POPTR | BBCH97 | 19 | 2.9 | <0.01 | 0.91 | 0.5 | 1.44 | 21 |
| POPTR | BBCH97 | 21 | -0.7 | ns | -0.42 | -1.57 | 1 | 14 |
| POPTR | BBCH97 | 22 | 0.8 | ns | 0.29 | -1 | 1.33 | 12 |
| POPTR | BBCH97 | 24 | -1.6 | ns | -0.63 | -2 | 0.5 | 13 |
| POPTR | BBCH97 | 30 | -1.3 | ns | -0.17 | -0.94 | 0.39 | 20 |
| POPTR | BBCH97 | 32 | -1.2 | ns | -0.42 | -1 | 0.3 | 18 |
| POPTR | BBCH97 | 34 | 0.5 | ns | 0.07 | -0.44 | 0.43 | 19 |
| POPTR | BBCH97 | 36 | -1.5 | ns | -0.44 | -1 | 0.22 | 15 |
| POPTR | BBCH97 | 37 | -0.2 | ns | -0.25 | -1.5 | 2 | 11 |
| POPTR | BBCH97 | 38 | 0.3 | ns | 0.11 | -0.36 | 0.73 | 20 |
| POPTR | BBCH97 | 39 | -1.9 | ns | -0.63 | -1.09 | 0.14 | 20 |
| POPTR | BBCH97 | 41 | -1.1 | ns | -0.6 | -1.33 | 0.5 | 16 |
| POPTR | BBCH97 | 42 | -3.4 | <0.01 | -1.16 | -2.33 | -0.58 | 13 |
| PRNPA | BBCH65 | 1 | -1.1 | ns | -0.48 | -1.17 | 0.38 | 21 |
| PRNPA | BBCH65 | 5 | -1 | ns | -0.6 | -3 | 1.25 | 10 |
| PRNPA | BBCH65 | 6 | -1.6 | ns | -0.5 | -2.8 | 0.5 | 11 |
| PRNPA | BBCH65 | 8 | -0.8 | ns | -0.37 | -1.14 | 0.4 | 20 |
| PRNPA | BBCH65 | 10 | -2 | ns | -0.6 | -1.08 | -0.08 | 20 |
| PRNPA | BBCH65 | 14 | -0.8 | ns | -0.25 | -1 | 0.44 | 19 |
| PRNPA | BBCH65 | 18 | -2.8 | <0.01 | -0.67 | -1.2 | -0.4 | 18 |
| PRNPA | BBCH65 | 19 | -1.1 | ns | -0.29 | -0.87 | 0.27 | 21 |
| PRNPA | BBCH65 | 21 | -0.7 | ns | -0.19 | -0.8 | 0.5 | 21 |
| PRNPA | BBCH65 | 22 | -1.9 | ns | -0.8 | -1.5 | 0.11 | 15 |

| | | | | | | | | |
|---|---|---|---|---|---|---|---|---|
| PRNPA | BBCH65 | 24 | -1.6 | ns | -0.81 | -2 | 0.5 | 13 |
| PRNPA | BBCH65 | 26 | -2.1 | <0.05 | -1.25 | -2.2 | -0.29 | 11 |
| PRNPA | BBCH65 | 28 | -0.8 | ns | -0.33 | -2 | 1 | 14 |
| PRNPA | BBCH65 | 29 | -1.8 | ns | -1.25 | -2 | 0.17 | 10 |
| PRNPA | BBCH65 | 30 | -1.1 | ns | -0.29 | -0.89 | 0.33 | 20 |
| PRNPA | BBCH65 | 31 | -2.6 | <0.05 | -0.57 | -1.06 | -0.17 | 15 |
| PRNPA | BBCH65 | 32 | -0.8 | ns | -0.2 | -0.94 | 0.64 | 19 |
| PRNPA | BBCH65 | 34 | 0 | ns | 0 | -0.65 | 0.39 | 20 |
| PRNPA | BBCH65 | 36 | -1 | ns | -0.22 | -0.86 | 0.39 | 19 |
| PRNPA | BBCH65 | 37 | -0.7 | ns | -0.75 | -3 | 1.4 | 11 |
| PRNPA | BBCH65 | 38 | -0.5 | ns | -0.09 | -0.8 | 0.5 | 20 |
| PRNPA | BBCH65 | 39 | 0.4 | ns | 0.15 | -0.46 | 0.53 | 20 |
| PRNPA | BBCH65 | 40 | -0.8 | ns | -0.7 | -3 | 1 | 11 |
| PRNPA | BBCH65 | 41 | 1.1 | ns | 0.33 | -0.33 | 1 | 19 |
| PRNPA | BBCH65 | 42 | 0 | ns | 0 | -0.56 | 0.5 | 19 |
| PRNPA | BBCH87 | 1 | -1.6 | ns | -1 | -2.25 | 0.5 | 14 |
| PRNPA | BBCH87 | 5 | -1.1 | ns | -0.71 | -1.5 | 0.67 | 10 |
| PRNPA | BBCH87 | 10 | 0.9 | ns | 0.67 | -1 | 2 | 11 |
| PRNPA | BBCH87 | 14 | 0.1 | ns | 0 | -0.89 | 1.5 | 16 |
| PRNPA | BBCH87 | 18 | -2.9 | <0.01 | -1.56 | -3 | -1 | 14 |
| PRNPA | BBCH87 | 19 | 0 | ns | 0 | -0.75 | 0.71 | 15 |
| PRNPA | BBCH87 | 21 | -0.9 | ns | -0.83 | -1.67 | 0.64 | 16 |
| PRNPA | BBCH87 | 22 | -2.3 | <0.05 | -1.71 | -2.82 | -0.25 | 14 |
| PRNPA | BBCH87 | 24 | 1 | ns | 0.93 | -1 | 2.25 | 13 |
| PRNPA | BBCH87 | 30 | -1.1 | ns | -0.5 | -1.13 | 0.83 | 19 |
| PRNPA | BBCH87 | 32 | -2.4 | <0.05 | -1.23 | -2.89 | -0.25 | 11 |
| PRNPA | BBCH87 | 34 | 0.8 | ns | 0.46 | -0.67 | 1.5 | 18 |
| PRNPA | BBCH87 | 36 | -1.6 | ns | -0.6 | -1.6 | 0.5 | 13 |
| PRNPA | BBCH87 | 37 | 0.3 | ns | 0.33 | -1.5 | 2.25 | 12 |
| PRNPA | BBCH87 | 38 | 0.5 | ns | 0.54 | -0.69 | 2 | 12 |
| PRNPA | BBCH87 | 39 | -0.5 | ns | -0.21 | -0.73 | 0.5 | 17 |
| PRNPA | BBCH87 | 41 | 1.3 | ns | 0.48 | -0.33 | 1 | 17 |
| SOUAU | BBCH07 | 1 | -2.7 | <0.01 | -1.07 | -2 | -0.4 | 15 |
| SOUAU | BBCH07 | 2 | -2 | ns | -1.75 | -3.2 | -0.33 | 10 |
| SOUAU | BBCH07 | 6 | -2.3 | <0.05 | -1.65 | -2.8 | -0.33 | 13 |
| SOUAU | BBCH07 | 8 | -0.9 | ns | -0.33 | -1.21 | 0.5 | 18 |
| SOUAU | BBCH07 | 10 | -0.9 | ns | -0.2 | -0.78 | 0.5 | 19 |
| SOUAU | BBCH07 | 14 | 0.2 | ns | 0.08 | -1 | 0.9 | 18 |
| SOUAU | BBCH07 | 18 | 0.7 | ns | 0.38 | -0.5 | 1.33 | 17 |
| SOUAU | BBCH07 | 19 | 1.4 | ns | 0.32 | -0.33 | 1 | 20 |
| SOUAU | BBCH07 | 21 | -0.6 | ns | -0.21 | -1 | 0.44 | 19 |
| SOUAU | BBCH07 | 22 | -0.9 | ns | -0.4 | -1.33 | 0.5 | 14 |
| SOUAU | BBCH07 | 24 | 0.2 | ns | 0.27 | -1 | 1 | 12 |
| SOUAU | BBCH07 | 26 | 1.1 | ns | 1 | -1 | 3.5 | 10 |
| SOUAU | BBCH07 | 28 | 1.1 | ns | 0.68 | -0.6 | 1.8 | 13 |
| SOUAU | BBCH07 | 30 | 0.9 | ns | 0.14 | -0.44 | 0.67 | 19 |
| SOUAU | BBCH07 | 31 | 0.9 | ns | 0.27 | -0.5 | 0.75 | 15 |
| SOUAU | BBCH07 | 32 | 1.5 | ns | 0.38 | -0.14 | 1 | 18 |

| | | | | | | | | |
|---|---|---|---|---|---|---|---|---|
| SOUAU | BBCH07 | 34 | -1.5 | ns | -0.54 | -1.38 | 0.29 | 19 |
| SOUAU | BBCH07 | 36 | -0.6 | ns | -0.13 | -0.71 | 0.33 | 18 |
| SOUAU | BBCH07 | 37 | 0.9 | ns | 0.14 | -0.6 | 1 | 11 |
| SOUAU | BBCH07 | 38 | 0.3 | ns | 0 | -0.5 | 0.78 | 19 |
| SOUAU | BBCH07 | 39 | 1.7 | ns | 0.38 | -0.3 | 1 | 18 |
| SOUAU | BBCH07 | 40 | -0.9 | ns | -0.29 | -2 | 1 | 11 |
| SOUAU | BBCH07 | 41 | 2.7 | <0.05 | 0.67 | 0.25 | 1.1 | 18 |
| SOUAU | BBCH07 | 42 | -2.1 | <0.05 | -0.86 | -1.75 | -0.13 | 19 |
| SOUAU | BBCH13 | 1 | -1.8 | ns | -0.56 | -1.25 | 0.13 | 20 |
| SOUAU | BBCH13 | 2 | -1.1 | ns | -1.5 | -5.5 | 1.5 | 10 |
| SOUAU | BBCH13 | 6 | -2.6 | <0.05 | -1.17 | -1.71 | -0.67 | 13 |
| SOUAU | BBCH13 | 8 | -1.7 | ns | -0.53 | -1.2 | 0.17 | 19 |
| SOUAU | BBCH13 | 10 | -0.2 | ns | 0 | -0.77 | 0.67 | 19 |
| SOUAU | BBCH13 | 14 | -0.7 | ns | -0.29 | -1 | 0.63 | 18 |
| SOUAU | BBCH13 | 18 | 0.5 | ns | 0.12 | -0.5 | 1 | 17 |
| SOUAU | BBCH13 | 19 | 0.1 | ns | 0 | -0.5 | 0.8 | 20 |
| SOUAU | BBCH13 | 21 | -1.3 | ns | -0.24 | -0.64 | 0.3 | 19 |
| SOUAU | BBCH13 | 22 | 0.5 | ns | 0.13 | -0.88 | 1.17 | 14 |
| SOUAU | BBCH13 | 24 | 1.3 | ns | 0.47 | -0.67 | 2.43 | 12 |
| SOUAU | BBCH13 | 26 | 0 | ns | 0 | -3 | 2 | 10 |
| SOUAU | BBCH13 | 28 | 0.2 | ns | 0.17 | -1 | 2 | 13 |
| SOUAU | BBCH13 | 30 | 0.9 | ns | 0.2 | -0.4 | 0.92 | 19 |
| SOUAU | BBCH13 | 31 | -0.1 | ns | 0 | -1 | 0.8 | 13 |
| SOUAU | BBCH13 | 32 | 0 | ns | 0 | -0.43 | 0.5 | 18 |
| SOUAU | BBCH13 | 34 | -2.1 | <0.05 | -0.33 | -0.83 | -0.07 | 19 |
| SOUAU | BBCH13 | 36 | -0.6 | ns | -0.14 | -0.67 | 0.6 | 17 |
| SOUAU | BBCH13 | 37 | 0.2 | ns | 0.17 | -1.43 | 1 | 10 |
| SOUAU | BBCH13 | 38 | 0.1 | ns | 0 | -0.5 | 0.6 | 19 |
| SOUAU | BBCH13 | 39 | 2.3 | <0.05 | 0.6 | 0.18 | 1.14 | 18 |
| SOUAU | BBCH13 | 40 | 0 | ns | 0 | -1.33 | 1.57 | 11 |
| SOUAU | BBCH13 | 41 | 0.8 | ns | 0.3 | -0.44 | 1 | 18 |
| SOUAU | BBCH13 | 42 | 0.8 | ns | 0.2 | -0.25 | 0.75 | 18 |
| SOUAU | BBCH65 | 1 | 0.3 | ns | 0.1 | -0.75 | 0.94 | 21 |
| SOUAU | BBCH65 | 2 | -2 | ns | -1.75 | -3.67 | -0.5 | 10 |
| SOUAU | BBCH65 | 3 | -2.4 | <0.05 | -1.5 | -3 | -0.33 | 10 |
| SOUAU | BBCH65 | 6 | 0 | ns | 0 | -1 | 1.5 | 13 |
| SOUAU | BBCH65 | 8 | 0.2 | ns | 0.07 | -0.67 | 0.89 | 19 |
| SOUAU | BBCH65 | 10 | -1.3 | ns | -0.49 | -1.11 | 0.42 | 20 |
| SOUAU | BBCH65 | 14 | -0.6 | ns | -0.29 | -1 | 0.64 | 19 |
| SOUAU | BBCH65 | 18 | -2 | ns | -0.77 | -1.25 | -0.07 | 18 |
| SOUAU | BBCH65 | 19 | -0.9 | ns | -0.29 | -0.89 | 0.33 | 20 |
| SOUAU | BBCH65 | 21 | -2 | ns | -0.5 | -1.13 | -0.06 | 20 |
| SOUAU | BBCH65 | 22 | -1.7 | ns | -0.54 | -1.14 | 0.14 | 15 |
| SOUAU | BBCH65 | 24 | -0.9 | ns | -0.24 | -1.33 | 0.63 | 13 |
| SOUAU | BBCH65 | 26 | -1.6 | ns | -0.86 | -2.67 | 0.5 | 11 |
| SOUAU | BBCH65 | 28 | -1 | ns | -0.58 | -1.25 | 0.8 | 13 |
| SOUAU | BBCH65 | 30 | -1.9 | ns | -0.43 | -1 | 0.09 | 19 |
| SOUAU | BBCH65 | 31 | -2.9 | <0.01 | -0.88 | -1.5 | -0.58 | 17 |

| | | | | | | | | |
|---|---|---|---|---|---|---|---|---|
| SOUAU | BBCH65 | 32 | -1.8 | ns | -0.5 | -1 | 0.17 | 19 |
| SOUAU | BBCH65 | 34 | -2.2 | <0.05 | -0.55 | -1 | -0.14 | 20 |
| SOUAU | BBCH65 | 36 | -0.9 | ns | -0.23 | -0.72 | 0.59 | 16 |
| SOUAU | BBCH65 | 38 | -1.6 | ns | -0.36 | -0.85 | 0.17 | 20 |
| SOUAU | BBCH65 | 39 | -1.4 | ns | -0.31 | -0.83 | 0.29 | 17 |
| SOUAU | BBCH65 | 41 | -1.1 | ns | -0.2 | -0.63 | 0.31 | 18 |
| SOUAU | BBCH65 | 42 | -1.2 | ns | -0.29 | -1 | 0.25 | 18 |
| SOUAU | BBCH87 | 1 | -0.7 | ns | -0.5 | -1.9 | 1 | 15 |
| SOUAU | BBCH87 | 3 | 0 | ns | -0.13 | -2.5 | 1.33 | 10 |
| SOUAU | BBCH87 | 5 | -0.9 | ns | -0.5 | -1.25 | 1 | 10 |
| SOUAU | BBCH87 | 6 | 1.3 | ns | 0.8 | -1.29 | 2.5 | 10 |
| SOUAU | BBCH87 | 8 | 0.7 | ns | 0.1 | -0.71 | 1.4 | 16 |
| SOUAU | BBCH87 | 10 | 1.8 | ns | 0.54 | -0.13 | 1.38 | 16 |
| SOUAU | BBCH87 | 14 | 1.3 | ns | 0.73 | -0.6 | 1.75 | 15 |
| SOUAU | BBCH87 | 18 | 1.3 | ns | 0.69 | -0.6 | 1.75 | 13 |
| SOUAU | BBCH87 | 19 | 2.2 | <0.05 | 0.7 | 0.17 | 1.5 | 19 |
| SOUAU | BBCH87 | 21 | 0.7 | ns | 0.22 | -0.63 | 1.43 | 16 |
| SOUAU | BBCH87 | 22 | -0.6 | ns | -0.18 | -1.5 | 1.09 | 13 |
| SOUAU | BBCH87 | 24 | 0.5 | ns | 0.4 | -1.57 | 2.5 | 11 |
| SOUAU | BBCH87 | 30 | 1.2 | ns | 0.3 | -0.33 | 0.86 | 20 |
| SOUAU | BBCH87 | 32 | 0.1 | ns | 0 | -0.78 | 0.82 | 14 |
| SOUAU | BBCH87 | 34 | -0.4 | ns | -0.23 | -1 | 0.8 | 16 |
| SOUAU | BBCH87 | 37 | -0.1 | ns | -0.25 | -2.5 | 1.5 | 10 |
| SOUAU | BBCH87 | 38 | 1.3 | ns | 0.89 | -0.17 | 2 | 15 |
| SOUAU | BBCH87 | 39 | -0.3 | ns | -0.1 | -1 | 0.67 | 15 |
| SOUAU | BBCH87 | 41 | 1.5 | ns | 1.11 | -0.5 | 1.8 | 14 |
| SOUAU | BBCH87 | 42 | -0.9 | ns | -0.38 | -2 | 1.25 | 11 |
| SOUAU | BBCH92 | 1 | 1.8 | ns | 0.68 | -0.11 | 1.5 | 20 |
| SOUAU | BBCH92 | 6 | 1.2 | ns | 0.67 | -1.14 | 2.5 | 13 |
| SOUAU | BBCH92 | 8 | 2.3 | <0.05 | 1.06 | 0.4 | 2.25 | 18 |
| SOUAU | BBCH92 | 10 | 2.7 | <0.05 | 1.59 | 0.67 | 3 | 16 |
| SOUAU | BBCH92 | 14 | 3 | <0.01 | 1.89 | 1 | 2.82 | 17 |
| SOUAU | BBCH92 | 18 | 2.8 | <0.01 | 1.5 | 0.69 | 3 | 15 |
| SOUAU | BBCH92 | 19 | 0.7 | ns | 0.39 | -0.5 | 1.26 | 19 |
| SOUAU | BBCH92 | 21 | 0.6 | ns | 0.14 | -0.5 | 0.67 | 15 |
| SOUAU | BBCH92 | 22 | 2.9 | <0.01 | 2.65 | 1 | 5 | 12 |
| SOUAU | BBCH92 | 24 | 1.6 | ns | 1.67 | -0.4 | 4 | 11 |
| SOUAU | BBCH92 | 30 | 3.6 | <0.001 | 1.61 | 0.89 | 2.5 | 17 |
| SOUAU | BBCH92 | 32 | 0.2 | ns | 0.07 | -0.83 | 1 | 16 |
| SOUAU | BBCH92 | 34 | 3.3 | <0.01 | 2.18 | 1 | 3.17 | 17 |
| SOUAU | BBCH92 | 36 | 0.5 | ns | 0.2 | -0.57 | 1.39 | 11 |
| SOUAU | BBCH92 | 37 | 1.9 | ns | 2 | -0.33 | 3.13 | 11 |
| SOUAU | BBCH92 | 38 | 2.8 | <0.01 | 1.16 | 0.5 | 2.14 | 17 |
| SOUAU | BBCH92 | 39 | 3 | <0.01 | 1 | 0.5 | 1.75 | 19 |
| SOUAU | BBCH92 | 41 | 2.4 | <0.05 | 0.79 | 0.39 | 2 | 14 |
| VACMY | BBCH65 | 1 | -1.1 | ns | -0.41 | -1.11 | 0.33 | 21 |
| VACMY | BBCH65 | 3 | -1.6 | ns | -0.67 | -1.27 | 0.22 | 19 |
| VACMY | BBCH65 | 5 | -0.7 | ns | -0.5 | -2.2 | 1.25 | 10 |

| | | | | | | | | |
|---|---|---|---|---|---|---|---|---|
| VACMY | BBCH65 | 6 | -1.9 | ns | -0.78 | -1.5 | 0.13 | 14 |
| VACMY | BBCH65 | 8 | -1.2 | ns | -0.4 | -1 | 0.27 | 21 |
| VACMY | BBCH65 | 10 | -2.1 | <0.05 | -0.45 | -1.2 | -0.14 | 20 |
| VACMY | BBCH65 | 14 | -1.2 | ns | -0.44 | -1 | 0.29 | 19 |
| VACMY | BBCH65 | 18 | -1.4 | ns | -0.35 | -1 | 0.25 | 18 |
| VACMY | BBCH65 | 19 | -2.1 | <0.05 | -0.67 | -1 | -0.13 | 21 |
| VACMY | BBCH65 | 21 | -0.7 | ns | -0.19 | -0.83 | 0.57 | 21 |
| VACMY | BBCH65 | 22 | -1.3 | ns | -1 | -1.67 | 0.4 | 15 |
| VACMY | BBCH65 | 24 | -0.4 | ns | -0.44 | -2.13 | 1.25 | 13 |
| VACMY | BBCH65 | 26 | -2 | ns | -1.5 | -2.71 | -0.17 | 11 |
| VACMY | BBCH65 | 28 | -1.2 | ns | -0.74 | -2 | 0.33 | 13 |
| VACMY | BBCH65 | 29 | -2.3 | <0.05 | -1.5 | -3.75 | -0.83 | 10 |
| VACMY | BBCH65 | 30 | -2 | ns | -0.49 | -1 | -0.08 | 20 |
| VACMY | BBCH65 | 31 | -1.5 | ns | -0.4 | -1.11 | 0.13 | 18 |
| VACMY | BBCH65 | 32 | -0.4 | ns | -0.11 | -0.83 | 0.63 | 19 |
| VACMY | BBCH65 | 34 | -2.5 | <0.05 | -0.5 | -0.93 | -0.18 | 19 |
| VACMY | BBCH65 | 36 | -1.1 | ns | -0.33 | -1 | 0.5 | 17 |
| VACMY | BBCH65 | 37 | -0.1 | ns | -0.17 | -2.5 | 2.25 | 10 |
| VACMY | BBCH65 | 38 | -0.7 | ns | -0.2 | -0.67 | 0.36 | 20 |
| VACMY | BBCH65 | 39 | -0.1 | ns | 0 | -0.6 | 0.57 | 21 |
| VACMY | BBCH65 | 40 | -1.3 | ns | -0.9 | -3.4 | 0.67 | 11 |
| VACMY | BBCH65 | 41 | 1.2 | ns | 0.33 | -0.5 | 0.88 | 19 |
| VACMY | BBCH65 | 42 | -0.8 | ns | -0.17 | -0.62 | 0.33 | 20 |
| VACMY | BBCH86 | 1 | 0.5 | ns | 0.19 | -0.61 | 1.46 | 21 |
| VACMY | BBCH86 | 2 | -0.1 | ns | 0 | -2 | 1.67 | 11 |
| VACMY | BBCH86 | 3 | -0.4 | ns | -0.06 | -1.33 | 0.92 | 20 |
| VACMY | BBCH86 | 5 | -1.3 | ns | -0.88 | -3.5 | 1 | 10 |
| VACMY | BBCH86 | 6 | 0.8 | ns | 0.33 | -1 | 1.38 | 14 |
| VACMY | BBCH86 | 8 | 2 | ns | 0.8 | 0.12 | 1.75 | 20 |
| VACMY | BBCH86 | 10 | -0.7 | ns | -0.2 | -0.86 | 0.43 | 18 |
| VACMY | BBCH86 | 14 | 0.8 | ns | 0.4 | -0.5 | 1 | 19 |
| VACMY | BBCH86 | 18 | 1.4 | ns | 0.55 | -0.33 | 1.86 | 16 |
| VACMY | BBCH86 | 19 | -0.4 | ns | -0.03 | -0.77 | 0.5 | 21 |
| VACMY | BBCH86 | 21 | 0.4 | ns | 0.13 | -0.5 | 0.67 | 20 |
| VACMY | BBCH86 | 22 | -1.6 | ns | -0.8 | -1.5 | 0.5 | 15 |
| VACMY | BBCH86 | 24 | -1.5 | ns | -0.54 | -1 | 0.33 | 13 |
| VACMY | BBCH86 | 26 | -0.3 | ns | -0.25 | -3 | 4 | 11 |
| VACMY | BBCH86 | 28 | -1.2 | ns | -0.88 | -1.88 | 0.78 | 11 |
| VACMY | BBCH86 | 30 | -3 | <0.01 | -0.45 | -0.79 | -0.25 | 20 |
| VACMY | BBCH86 | 31 | -1.6 | ns | -0.32 | -1 | 0.25 | 16 |
| VACMY | BBCH86 | 32 | 0.3 | ns | 0.11 | -0.67 | 1 | 18 |
| VACMY | BBCH86 | 34 | -1.8 | ns | -0.4 | -0.75 | 0.2 | 19 |
| VACMY | BBCH86 | 36 | -0.3 | ns | -0.11 | -1 | 0.67 | 18 |
| VACMY | BBCH86 | 37 | 0 | ns | 0 | -1 | 1 | 10 |
| VACMY | BBCH86 | 38 | 1.3 | ns | 0.43 | -0.25 | 1.17 | 20 |
| VACMY | BBCH86 | 39 | 0.3 | ns | 0.06 | -0.44 | 0.67 | 21 |
| VACMY | BBCH86 | 40 | -0.9 | ns | -0.67 | -2.83 | 2 | 11 |
| VACMY | BBCH86 | 41 | 1.7 | ns | 0.88 | -0.15 | 1.78 | 18 |

| | | | | | | | | |
|---|---|---|---|---|---|---|---|---|
| VACMY | BBCH86 | 42 | -0.1 | ns | 0 | -0.31 | 0.73 | 18 |
| VACMY | BBCH87 | 1 | -0.9 | ns | -0.4 | -1 | 0.35 | 20 |
| VACMY | BBCH87 | 2 | -1.6 | ns | -0.5 | -3.4 | 1.38 | 11 |
| VACMY | BBCH87 | 3 | 0.1 | ns | 0.08 | -0.81 | 1 | 21 |
| VACMY | BBCH87 | 5 | -1.2 | ns | -0.89 | -3.5 | 1.25 | 10 |
| VACMY | BBCH87 | 6 | 0.3 | ns | 0.2 | -1 | 1.25 | 14 |
| VACMY | BBCH87 | 8 | 1 | ns | 0.5 | -0.47 | 1.17 | 21 |
| VACMY | BBCH87 | 10 | -1.3 | ns | -0.38 | -0.9 | 0.25 | 14 |
| VACMY | BBCH87 | 14 | 0.9 | ns | 0.45 | -0.6 | 1.4 | 16 |
| VACMY | BBCH87 | 18 | 0 | ns | 0 | -0.86 | 0.83 | 16 |
| VACMY | BBCH87 | 19 | 0.2 | ns | 0.04 | -0.5 | 0.82 | 20 |
| VACMY | BBCH87 | 21 | -0.5 | ns | -0.17 | -0.6 | 0.6 | 20 |
| VACMY | BBCH87 | 22 | -0.8 | ns | -0.27 | -1.38 | 0.71 | 15 |
| VACMY | BBCH87 | 24 | 1.5 | ns | 0.38 | -0.33 | 1.29 | 13 |
| VACMY | BBCH87 | 26 | -1.3 | ns | -1.25 | -2.5 | 1.33 | 10 |
| VACMY | BBCH87 | 28 | 0.9 | ns | 0.43 | -1 | 2.25 | 11 |
| VACMY | BBCH87 | 30 | -0.6 | ns | -0.23 | -0.83 | 0.36 | 20 |
| VACMY | BBCH87 | 31 | -0.5 | ns | -0.14 | -0.91 | 0.5 | 15 |
| VACMY | BBCH87 | 32 | -0.5 | ns | -0.17 | -0.82 | 0.56 | 19 |
| VACMY | BBCH87 | 34 | -1 | ns | -0.36 | -0.83 | 0.33 | 19 |
| VACMY | BBCH87 | 36 | -1.4 | ns | -0.45 | -1.2 | 0.29 | 16 |
| VACMY | BBCH87 | 37 | 0.2 | ns | 0.2 | -1.33 | 2 | 11 |
| VACMY | BBCH87 | 38 | 0.1 | ns | 0 | -0.58 | 0.81 | 20 |
| VACMY | BBCH87 | 39 | -0.5 | ns | -0.1 | -0.6 | 0.46 | 21 |
| VACMY | BBCH87 | 41 | 0.3 | ns | 0.13 | -0.67 | 1.25 | 14 |
| VACMY | BBCH87 | 42 | -1.5 | ns | -0.33 | -1 | 0.29 | 17 |
| VACVI | BBCH65 | 1 | 0.9 | ns | 0.17 | -0.5 | 0.92 | 21 |
| VACVI | BBCH65 | 2 | -1.6 | ns | -1 | -2.2 | 0.5 | 11 |
| VACVI | BBCH65 | 3 | 0.9 | ns | 0.26 | -0.71 | 0.78 | 20 |
| VACVI | BBCH65 | 5 | -0.6 | ns | -0.6 | -4 | 1.25 | 10 |
| VACVI | BBCH65 | 6 | 0.1 | ns | 0.08 | -1.33 | 1.6 | 13 |
| VACVI | BBCH65 | 8 | 0.6 | ns | 0.15 | -0.67 | 1 | 20 |
| VACVI | BBCH65 | 10 | -2.3 | <0.05 | -0.67 | -1.58 | -0.15 | 17 |
| VACVI | BBCH65 | 14 | 0.9 | ns | 0.28 | -0.53 | 0.89 | 19 |
| VACVI | BBCH65 | 18 | -1.3 | ns | -0.6 | -1.38 | 0.4 | 18 |
| VACVI | BBCH65 | 19 | -1.1 | ns | -0.37 | -1 | 0.33 | 21 |
| VACVI | BBCH65 | 21 | -2.2 | <0.05 | -0.71 | -1.31 | -0.2 | 21 |
| VACVI | BBCH65 | 22 | -1.5 | ns | -0.64 | -1.43 | 0.38 | 15 |
| VACVI | BBCH65 | 24 | -1 | ns | -0.32 | -1.25 | 0.5 | 13 |
| VACVI | BBCH65 | 26 | -1.1 | ns | -0.29 | -2.14 | 1 | 11 |
| VACVI | BBCH65 | 28 | -0.6 | ns | -0.63 | -2.2 | 1 | 11 |
| VACVI | BBCH65 | 30 | -2.2 | <0.05 | -0.49 | -0.88 | -0.15 | 20 |
| VACVI | BBCH65 | 31 | -3.3 | <0.01 | -0.77 | -1.33 | -0.5 | 18 |
| VACVI | BBCH65 | 32 | -0.9 | ns | -0.38 | -1.13 | 0.58 | 17 |
| VACVI | BBCH65 | 34 | -1.1 | ns | -0.36 | -1.18 | 0.63 | 18 |
| VACVI | BBCH65 | 36 | -2.8 | <0.01 | -0.8 | -1.33 | -0.27 | 17 |
| VACVI | BBCH65 | 37 | 1.3 | ns | 0.5 | -0.56 | 1.4 | 11 |
| VACVI | BBCH65 | 38 | -0.5 | ns | -0.17 | -0.67 | 0.44 | 20 |

| | | | | | | | | |
|---|---|---|---|---|---|---|---|---|
| VACVI | BBCH65 | 39 | -1.3 | ns | -0.34 | -0.71 | 0.24 | 21 |
| VACVI | BBCH65 | 40 | -0.7 | ns | -0.4 | -2.5 | 1 | 11 |
| VACVI | BBCH65 | 41 | 0.9 | ns | 0.31 | -0.4 | 0.9 | 18 |
| VACVI | BBCH65 | 42 | -2.4 | <0.05 | -0.36 | -0.71 | -0.13 | 18 |
| VACVI | BBCH86 | 1 | 1.6 | ns | 1 | -0.33 | 2.25 | 19 |
| VACVI | BBCH86 | 2 | -0.5 | ns | -0.5 | -2.17 | 2.14 | 11 |
| VACVI | BBCH86 | 3 | 1.7 | ns | 0.93 | -0.33 | 2 | 19 |
| VACVI | BBCH86 | 5 | -0.1 | ns | 0 | -2 | 2 | 10 |
| VACVI | BBCH86 | 6 | 2.4 | <0.05 | 2.27 | 0.5 | 3.83 | 13 |
| VACVI | BBCH86 | 8 | 1.3 | ns | 0.53 | -0.78 | 1.44 | 20 |
| VACVI | BBCH86 | 10 | 0.7 | ns | 0.33 | -0.91 | 1.5 | 15 |
| VACVI | BBCH86 | 14 | 2.5 | <0.05 | 1.18 | 0.33 | 1.93 | 17 |
| VACVI | BBCH86 | 18 | 2.1 | <0.05 | 0.77 | 0.11 | 1.67 | 15 |
| VACVI | BBCH86 | 19 | 2.6 | <0.05 | 0.97 | 0.29 | 1.67 | 21 |
| VACVI | BBCH86 | 21 | 0.7 | ns | 0.2 | -0.4 | 0.75 | 21 |
| VACVI | BBCH86 | 22 | 0.5 | ns | 0.46 | -0.5 | 1.2 | 14 |
| VACVI | BBCH86 | 24 | 0.2 | ns | 0.24 | -1 | 2 | 13 |
| VACVI | BBCH86 | 28 | -0.2 | ns | -0.33 | -1.6 | 3 | 10 |
| VACVI | BBCH86 | 30 | -0.9 | ns | -0.33 | -1 | 0.46 | 20 |
| VACVI | BBCH86 | 31 | -0.3 | ns | -0.13 | -1 | 0.8 | 15 |
| VACVI | BBCH86 | 32 | 1.3 | ns | 0.46 | -0.38 | 1.46 | 18 |
| VACVI | BBCH86 | 34 | 0.3 | ns | 0.14 | -0.8 | 1.31 | 18 |
| VACVI | BBCH86 | 36 | 0 | ns | 0 | -1.14 | 1 | 14 |
| VACVI | BBCH86 | 37 | 0.4 | ns | 0.5 | -1.22 | 2.33 | 11 |
| VACVI | BBCH86 | 38 | 0.8 | ns | 0.2 | -0.38 | 0.71 | 20 |
| VACVI | BBCH86 | 39 | -0.1 | ns | 0 | -0.67 | 0.8 | 21 |
| VACVI | BBCH86 | 41 | 1.5 | ns | 0.5 | -0.25 | 1.5 | 18 |
| VACVI | BBCH86 | 42 | -1 | ns | -0.29 | -1 | 0.5 | 15 |
| VACVI | BBCH87 | 1 | 1.2 | ns | 0.47 | -0.5 | 1.25 | 20 |
| VACVI | BBCH87 | 3 | 1.1 | ns | 0.33 | -0.5 | 1.27 | 20 |
| VACVI | BBCH87 | 5 | 1.5 | ns | 1.25 | -0.71 | 2.33 | 10 |
| VACVI | BBCH87 | 6 | 2.7 | <0.05 | 1.14 | 0.38 | 1.9 | 14 |
| VACVI | BBCH87 | 8 | -0.5 | ns | -0.3 | -1.31 | 1 | 20 |
| VACVI | BBCH87 | 10 | 1.4 | ns | 0.57 | -0.29 | 1.27 | 17 |
| VACVI | BBCH87 | 14 | -0.2 | ns | -0.18 | -1.29 | 1 | 17 |
| VACVI | BBCH87 | 18 | 1.8 | ns | 0.5 | -0.29 | 1.57 | 15 |
| VACVI | BBCH87 | 19 | 1.9 | ns | 0.73 | -0.07 | 1.57 | 20 |
| VACVI | BBCH87 | 21 | 0.2 | ns | 0.04 | -0.7 | 0.75 | 21 |
| VACVI | BBCH87 | 22 | 0.3 | ns | 0.14 | -1 | 1.25 | 15 |
| VACVI | BBCH87 | 24 | 0.9 | ns | 0.45 | -1 | 1.25 | 13 |
| VACVI | BBCH87 | 30 | 0.5 | ns | 0.15 | -0.53 | 0.8 | 20 |
| VACVI | BBCH87 | 31 | -0.1 | ns | -0.08 | -0.8 | 1 | 15 |
| VACVI | BBCH87 | 32 | -0.5 | ns | -0.18 | -0.79 | 0.5 | 18 |
| VACVI | BBCH87 | 34 | -0.9 | ns | -0.55 | -1.36 | 0.75 | 18 |
| VACVI | BBCH87 | 36 | 1.3 | ns | 0.44 | -0.21 | 1.07 | 15 |
| VACVI | BBCH87 | 37 | 0.7 | ns | 0.5 | -0.71 | 2 | 13 |
| VACVI | BBCH87 | 38 | 0.2 | ns | 0 | -0.75 | 1 | 17 |
| VACVI | BBCH87 | 39 | 1.8 | ns | 0.48 | -0.1 | 0.78 | 21 |

| | | | | | | | | |
|---|---|---|---|---|---|---|---|---|
| VACVI | BBCH87 | 41 | 0.6 | ns | 0.22 | -1 | 1.6 | 15 |
| VACVI | BBCH87 | 42 | 0.4 | ns | 0.08 | -0.74 | 1.18 | 15 |
